# Molecular Phylogeography Analysis Reveals Population Dynamics and Genetic Divergence of a Widespread Tree *Pterocarya stenoptera* in China

**DOI:** 10.3389/fgene.2019.01089

**Published:** 2019-11-01

**Authors:** Zhi-Hao Qian, Yong Li, Ming-Wan Li, Yan-Xia He, Jia-Xin Li, Xiao-Fan Ye

**Affiliations:** ^1^Innovation Platform of Molecular Biology, College of Forestry, Henan Agricultural University, Zhengzhou, China; ^2^School of Life Sciences, Henan University, Kaifeng, China

**Keywords:** cpDNA, microsatellite marker, phylogeography, *Pterocarya stenoptera*, species distribution modeling

## Abstract

The geological events, past climatic fluctuations, and river systems played key roles in the spatial distribution, population dynamics, and genetic differentiation of species. In this work, we selected *Pterocarya stenoptera*, a widespread tree species in China, to test the roles of these factors. Four noncoding spacers, eight microsatellite (simple sequence repeat) markers, and species distribution modeling were used to examine the phylogeographical pattern of *P. stenoptera*. Based on chloroplast DNA data, populations of *P. stenoptera* were clearly clustered into three groups. The divergence time of these groups fell into the stage of the Qinghai–Tibet Movement, 1.7–2.6 Ma. For simple sequence repeat data, only one western marginal population YNYB could be separated from other populations, whereas other populations were mixed together. Our results indicated that the environmental heterogeneity resulting from the Qinghai–Tibet movement might be response for this genetic divergence. The climatic fluctuations in the Pleistocene did not cause the substantial range shift of *P. stenoptera*, while the fluctuations affected its population size. Moreover, we also confirmed the river systems did not act as channels or barrier of dispersal for *P. stenoptera*.

## Introduction

The uplift of the Qinghai–Tibet Plateau (QTP) has not only changed the topography of the area and its surroundings but also played a remarkable role in the climate change of Asia (An et al., 2006; Deng and Ding, 2015). The complex terrain and climate caused by this uplift exacerbated the rapid differentiation of species on the plateau, thus transforming the QTP and its adjacent areas into one of the world’s richest regions in China (Wu, 1987). Furthermore, the uplift of the QTP caused a profound impact on species differentiation in other regions of Asia. The fast uplift of the QTP, i.e., the Qinghai–Tibet movement (since ca. 3.6 Ma), caused a remarkable increase in the eolian flux in the north Pacific, and this increase in eolian flux has remained steady until today (Li et al., 1979; Rea et al., 1998). As a consequence of this change, the Asian inland began to experience aridification, while southeast China became increasingly humid (Li et al., 2015). To date, little attention has been paid to whether climate change in Asia caused by the Qinghai–Tibet movement has affected species differentiation (Liu et al., 2013). Most of the research attention has focused on the impact of the recent uplift of the QTP, i.e., Kunlun–Yellow River movement (Liu et al., 2012). This movement, which occurred approximately 1.1–0.7 Ma, affected the whole QTP and lifted the plateau surface to 3,000–3,500 m (Cui et al., 1998; Shi, 1998). Even some areas of the mountain ranges were uplifted to a height of 4,500–5,000 m, thus allowing the development of mountain glaciers in response to global glacial–interglacial cycles (Shi, 1998; Zhou et al., 2006). The movement also influenced the pattern of atmospheric circulation and formed the modern pattern of the East Asian monsoon (Jiang et al., 2005). Most of East Asia was not covered by glaciers, but the mountain glacial cycles after the Kunlun–Yellow River movement notably caused the climatic fluctuation in this area (Shi et al., 2006). The climatic oscillations during the Pleistocene substantially shaped the spatial distribution, population dynamics, and genetic differentiation of species (Comes and Kadereit, 1998; Hewitt, 2000; Hewitt, 2004).

Molecular phylogeographical methods seek to unravel historic biogeographical processes in response to climatic oscillations during the Pleistocene (Petit et al., 2003; Avise, 2009; Hickerson et al., 2010). The rise in phylogeographical studies for an increasing number of species and the resultant accumulated evidence suggest that the species have responded to climate fluctuations in two ways (Fu et al., 2014; Miao et al., 2017a). The first type of response is the adjustment in distribution range of these species when they migrated southward or to main refugia during glaciation and their recolonization after glaciation; the other type is the persistence *in situ* in the form of local adaptation during multiple glacial–interglacial cycles (Li et al., 2017; Miao et al., 2017b). Response modes are determined by the tolerance and migration capacity of species. However, regardless of the response patterns, species population dynamics are more likely affected by the Pleistocene climatic fluctuations. The glacial–interglacial cycle (0.7 Ma to the present), which was strengthened by the Kunlun–Yellow River movement, occurred many times in East Asia (Cui et al., 2011). Which cycle has the greatest impact on species population dynamics in this region during the Pleistocene?

*Pterocarya stenoptera* C. DC (Juglandaceae) is a dominant deciduous broad-leaved tree growing in forests below 1,500 m above sea level. The species is widely distributed in the warm-temperate and subtropical regions in China. *P. stenoptera* is intolerant of cold and drought, and it is likely to grow in warm and humid environments (Xu et al., 2002). Flowers bloom in April and May, and fruit ripening extends to August and September. The samara of *P. stenoptera* becomes dry after ripening and can be dispersed by wind and water flow. According to our field observations, *P. stenoptera* is mainly distributed along stream banks. Thus, knowing whether rivers have an impact on genetic patterns of *P. stenoptera* is worth discussing. The three relatively large rivers in the distribution area of *P. stenoptera* are the Yellow River, the Huaihe River, and the Yangtze River. In previous phylogeographical studies, rivers serve as physical barriers or dispersal channels (Zhang et al., 2011; Wang et al., 2014; Geng et al., 2015; Cazé et al., 2016; Liang et al., 2018). *P. stenoptera*, as a dominant deciduous broad-leaved tree of China, is therefore an ideal candidate in the investigation of the influences of geological events, climate change, and river systems on the genetic differentiation and population dynamics of species in this region.

Here, we employed chloroplast DNA (cpDNA) noncoding spacers, microsatellite [simple sequence repeat (SSR)] markers, and species distribution modeling (SDM) to investigate the phylogeographical pattern of *P. stenoptera*. Our specific aims were to address two main questions: (i) What is the genetic structure of *P. stenoptera* populations in China as revealed by cpDNA data and SSR markers? (ii) How do geological events, climate change, and river systems affect the genetic differentiation and population dynamics of *P. stenoptera*?

## Materials and Methods

### Population Sampling

**Figure 1:**
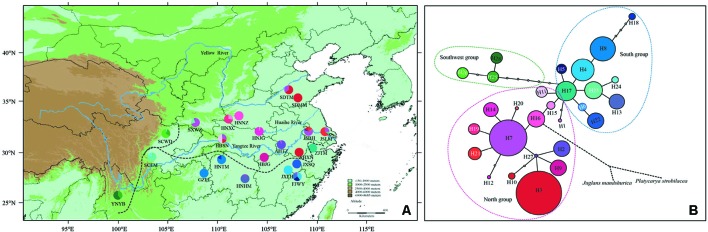
Geographic distribution and genealogical relationships of chloroplast DNA (cpDNA) haplotypes recovered from *Pterocarya stenoptera* populations in China. **(A)** Sampling localities of 22 populations of *P. stenoptera* and the geographic distribution of 28 haplotypes (H1–H28) detected (for population codes see **Table 1**). The dotted line represents the dividing line of three genetic groups that identified by cpDNA data. **(B)** The statistical parsimony network of haplotypes H1–H28. The size of circles corresponds to the frequency of each haplotype. Each solid line represents one mutational step interconnecting two haplotypes for which parsimony is supported at the 95% level, the black dotted line represents multiple mutational steps that connected with outgroups. The colorful dotted line represents the dividing line of three genetic groups that identified by cpDNA data.

Fresh leaves of 440 samples of *P. stenoptera* distributed in 22 localities were collected covering its entire distribution area in China during May to November 2017 (**Figure 1** and **Table 1**). Each population collects 20 individuals, and they are at least 20 m apart. The leaves were dried immediately in silica gel and stored at room temperature. The geographical information of these populations and numbers of individuals used in the cpDNA and SSR analyses were presented in **Table 1**. No specific permits are required for the collection of *P. stenoptera*, and all samples were collected under current national and local regulations.

**Table 1:** Details of population locations, sample size, cpDNA, and SSR variation of *Pterocarya stenoptera* sampled in China.

Population no. and code	Locations	Lat.(N)/Long.(E)	cpDNA					SSR			
			N	Haplotypes (no. of individuals)	π × 10^−3^	*h*	*N*_Ph_	*N*	*H*_E_	*A*_R_	*N*_PA_
North group											
1.SDTM	Tai Mt., Shandong	36.22/117.12	20	H3(13), H7(7)	0.43	0.479	0	20	0.270	1.931	0
2.SDMM	Meng Mt., Shandong	35.56/117.96	20	H3(20)	0	0	0	20	0.323	2.528	4
3.HNNZ	Nanzhao, Henan	33.59/112.18	20	H16(20)	0	0	0	20	0.353	2.749	0
4.HNXC	Xichuan, Henan	33.28/111.12	20	H15(5), H16(1), H19(14)	0.58	0.468	1	20	0.338	2.730	0
5.SXWZ	Wuzi Mt., Shaanxi	32.95/107.84	20	H7(14), H23(5), H27(1)	0.73	0.468	1	20	0.354	2.174	0
6.JSBH	Baohua Mt., Jiangshu	32.14/119.09	20	H3(3), H5(1), H7(12), H20(1), H21(3)	0.58	0.621	1	20	0.365	2.771	0
7.JSLM	Lang Mt., Jiangshu	31.95/120.89	20	H7(5), H17(2), H21(13)	0.47	0.532	0	20	0.280	2.119	0
8.HNJG	Jigong Mt., Henan	31.81/114.08	20	H7(1), H14(18), H15(1)	0.18	0.195	1	20	0.337	2.755	0
9.HBSN	Shengnongjia, Hubei	31.37/110.50	20	H2(1), H7(5), H10(4), H11(10)	1.24	0.679	2	20	0.342	2.768	0
10.AHTZ	Tianzhu Mt., Anhui	30.67/116.49	20	H1(1), H2(19)	0.14	0.100	1	20	0.343	2.687	2
11.AHXN	Xiuning, Anhui	29.78/118.17	20	H3(20)	0	0	0	20	0.381	2.438	0
12.HBJG	Jiugong Mt., Hubei	29.45/114.71	20	H9(20)	0	0	1	20	0.397	2.841	1
South group											
13.ZJTM	Tianmu Mt., Zhejiang	30.28/119.46	20	H17(20)	0	0	0	20	0.279	2.314	0
14.SCEM	Emei Mt., Sichuan	29.57/103.44	20	H23(16), H24(4)	0.15	0.337	1	20	0.363	2.422	0
15.HNTM	Tianmeng Mt., Hunan	29.11/110.46	20	H8(13), H17(3), H18(4)	0.55	0.542	1	20	0.371	2.740	0
16.JXSQ	Shanqing Mt., Jiangxi	28.84/118.04	20	H22(20)	0	0	1	20	0.291	2.042	0
17.JXLH	Longhu Mt., Jiangxi	28.12/116.97	20	H4(20)	0	0	0	20	0.194	2.196	0
18.GZFJ	Fengjing Mt., Guizhou	27.84/108.77	18	H8(18)	0	0	0	20	0.337	2.248	1
19.FJWY	Wuyi Mt., Fujian	27.65/117.97	20	H4(8), H5(5), H6(5), H7(2)	0.77	0.742	0	20	0.249	2.138	0
20.HNHM	Heng Mt., Hunan	27.26/112.72	20	H12(1), H13(19)	0.14	0.100	2	20	0.383	2.619	0
Southwest group											
21.SCWD	Wuduzhen, Sichuan	31.88/104.78	20	H23(1), H25(6), H26(13)	0.75	0.511	1	20	0.326	2.101	1
22.YNYB	Yangbi, Yunnan	25.62/100.03	20	H25(8), H28(12)	0.23	0.505	1	20	0.274	2.842	1

N, number of individuals; h, haplotype diversity; π, nucleotide diversity; N_Ph_, number of private haplotypes; H_E_, Nei’s (1987) measure of gene diversity; A_R_, Allelic richness; N_PA_, number of private alleles.

### DNA Isolation, SSR Genotyping, and DNA Sequencing

Total genomic DNA was extracted from 30 mg of silica-dried leaves using a DNA Extraction Kit DP305 (Tiangen, Beijing, China). Four noncoding cpDNA spacers of *ndh*Ax1-*ndh*Ax2, *rp*L34-*rp*L36, *trn*L(UAG)-*rp*L32-F (Shaw et al., 2007), and *trn*V(UAC)x2-*ndh*C (5′-TGCATGTTGGGTCTTTGA, 5′-CATCGCCCATTGGTTCTA) and eight microsatellite markers (Ps23, Ps28, Ps59, Ps69, Ps70, Ps75, Ps82, and Ps83) (Wang et al., 2018) were amplified for 438 and 440 samples respectively (**Table 1**). For cpDNA fragments, all the polymerase chain reactions (PCRs) were performed in a total volume of 40 µl, containing 30 ng of template DNA, 10 mM Taq buffer, 0.2 mM dNTPs, 0.3 mM primers, and 2 units of Taq polymerase (Tiangen, Beijing, China). PCRs were performed in a Mastercycler nexus thermocycler (Eppendorf, Hamburg, Germany). The PCR program was set to: an initial denaturation at 95°C for 5 min, 35 cycles of denaturation at 95°C for 40 s, 40 s of annealing at 52–60°C and 1 min at 72°C, and a final extension of 72°C for 5 min. The PCR products were sequenced using an ABI 3730 DNA Sequencer. The quality of original chromatogram of sequence was checked and aligned using CLUSTAL_X version 2.1 (Larkin et al., 2007). For SSR markers, PCRs were performed in a 20-µl reaction mixture consisting of 20 ng genomic DNA, 10 mM Taq buffer, 0.2 mM dNTPs, 0.3 mM primers, and 1 unit of Taq polymerase. The forward primers of SSRs were 5′ labeled with FAM, HEX, or TAMARA according to Wang et al. (2018). The PCR program was set to: an initial denaturation at 95°C for 5 min, 35 cycles of denaturation at 95°C for 40 s, 40 s of annealing at 48–62°C and 40 s at 72°C, and a final extension of 72°C for 5 min. PCR products were mixed with 10 µl of HiDi formamide and 0.1 µl of internal standard ROX500 (Applied Biosystems, Foster City, USA). These products were genotyped on an ABI 3730 DNA Analyzer.

## Data Analysis

### cpDNA Data

The cpDNA sequences of each noncoding spacer were aligned by CLUSTAL_X version 2.1 (Larkin et al., 2007). The indels in the cpDNA sequences were encoded as described by Caicedo and Schaal (2004). The genetic parameters of haplotype diversity (*h*) and nucleotide diversity (*π*) were estimated using dnasp version 6.12 (Rozas et al., 2017). The number of private haplotypes (*N*_Ph_) was counted by manual statistics. The networks of cpDNA haplotypes were constructed using TCS version 1.21 (Clement et al., 2000) with >20-step limitation. Divergence time, which was estimated by BEAST version 2.5.0 (Bouckaert et al., 2014), was used to relate the genetic differentiation of cpDNA lineages to the historic events of this species. The statistical parsimony network of 28 haplotypes were constructed. According to Zhang et al. (2013), *Juglans mandshurica* is a species close to *P. stenoptera*, while *Platycarya strobilacea* is a relatively distant species. Thus, we selected these two species as the outgroups before running BEAST module, and the best fit nucleotide substitution model of Hasegawa, Kishino, and Yano (HKY) was selected in MEGA version 10.0 (Kumar et al., 2018) in accordance with the Bayesian information criterion. Dating analysis was performed with a lognormal relaxed molecular clock model, four gamma categories, the Yule process of tree prior, HKY as site model, and a normal prior distribution for age constraints. Normal distribution prior root sets were used for the node ages to unite *J. mandshurica* = 39.72 Ma and *P. strobilacea* = 63.05 Ma in secondary calibration based on the results of Zhang et al. (2013). The sequences of *J. mandshurica* were deposited in GenBank with accession numbers MK002388, MK002394, MK002404, and MK002416. The sequences of *P. strobilacea* were downloaded from GenBank with accession number KX868670. We set the Markov chain Monte Carlo (MCMC) chain to 10 million generations at the sampling frequency of 1,000 generations each time. Tracer version 1.7.1 was used to visualize and check for convergence to a stationary distribution and ensure effective sampling size values (ESSs > 200). We discarded the first 10% as burn-in and constructed the maximum clade credibility tree by using TreeAnnotator version 2.5.1 (Bouckaert et al., 2014). The resulting tree, with ages appropriated to each node and 95% credibility intervals set as the divergence times, was drawn in FigTree version 1.4.3 (Rambaut, 2016). The genetic relationships of 22 populations of *P. stenoptera* were constructed the basis of their pairwise genetic distances in cpDNA and determined from the net average between populations of sequences (*D*_A_) by using the unweighted pair-group method with arithmetic module of MEGA version 10.0 (Kumar et al., 2018). The *D*_A_ value was estimated using MEGA version 10.0 (Kumar et al., 2018) under the maximum composite likelihood model (Tamura et al., 2004).

We retraced the demographic history of *P. stenoptera* by using the extended Bayesian skyline plot (EBSP) in BEAST version 2.5.0 to further infer potential relatively complex changes given an effective population size (Bouckaert et al., 2014). EBSP analyses were performed with a lognormal relaxed molecular clock model, HKY as the site model, and a normal prior distribution for age constraints. A normal distribution prior root set was used for the node-age results of the above calculations. The scale factor of 0.5 for cpDNA was adopted because only the female cpDNA contributes to effective population size, and then the MCMC chain was run in 10 million generations at a frequency of 1,000 generations each time.

Inferences for the historical demographic processes were generated by calculating Tajima’s *D* values (Tajima, 1989) and Fu’s *F*s values (Fu, 1997) for the total level and for each group in arlequin version 3.5.1 (Excoffier and Lischer, 2010), respectively. f a demographic expansion hypothesis is true, then significant negative values of Tajima’s *D* and Fu’s *F*s statistics are expected. Inferences for the demographic processes were generated by mismatch distribution analyses under the null hypotheses of the sudden expansion model in arlequin version 3.5.1 (Excoffier and Lischer, 2010).

### SSR Data

SSR alleles were identified on the basis of viewable peaks in GeneMarker version 2.2.0 (SoftGenetics, State College, Pennsylvania, USA). Nei’s gene diversity (*H*_E_; Nei, 1987), allele frequencies, and allelic richness (*A*_R_) were calculated by FSTAT version 2.9.3 (Goudet, 1995). The number of private alleles (*N*_PA_) was manually counted according to the allele frequencies in FSTAT. The role of rivers in the transmission of *P. stenoptera* was explored by performing correlation analyses between the level of genetic diversity based on SSR and cpDNA and longitude using the Mantel test in vegan package (Oksanen et al., 2017). Similarly, correlation analyses between the level of genetic diversity based on SSR and cpDNA and latitude were also performed using the Mantel test in vegan package. Bayesian analysis was performed in structure version 2.3.4 (Pritchard et al., 2000) to infer the most likely number of genetic subgroups (*K*) in the SSR dataset. The program was run with *K* values from 1 to 10 in 10 replicates for each *K*, 20,000 burn-in periods and 100,000 MCMC iterations. An admixture model with correlated allele frequencies was chosen for this analysis. The optimal *K* value was determined by computing the *∆K* values according to the method of Evanno et al. (2005); this method was executed in Structure Harvester (Earl and vonHoldt, 2012). The best *K* value was further estimated according to the value of the TI posterior probability by using RmavericK (Verity and Nichols, 2016). The average value of the admixture coefficients over 10 runs was calculated using CLUMPP version 1.1 (Jakobsson and Rosenberg, 2007). The bar plots of STRUCTURE were produced by DISTRUCT 1.1 (Rosenberg, 2004). A principal component analysis of the SSR data was performed using ggord package (Beck, 2017). For both SSR and cpDNA data, tests of isolation by distance (IBD) were performed by regressing the standardized values of *F*_ST_ against geographical distance (m) and the ecological distance *via* the Mantel permutation procedure in vegan package (Oksanen et al., 2017). The values of *F*_ST_ based on the SSR and cpDNA data were estimated by FSTAT and dnasp. Geographical distance was calculated by the geosphere package (Hijmans, 2015), and environmental distances were calculated as Euclidian distances along the principal components (PCs) according to the method of Pluess et al. (2016). The first four PCs can explain 91.11% of the variation for the present extracted 19 environmental variables. Nineteen environmental variables at 2.5 arcmin resolution for the present time were downloaded from the WorldClim website.

Genetic differentiations of cpDNA data and SSR data among populations and for populations within each group (*F*_ST_) were calculated by nonhierarchical analyses of molecular variance (AMOVAs) in ARLEQUIN version 3.5.1 (Excoffier and Lischer, 2010). The amount of genetic variations among groups, among populations within groups, and within populations was estimated with hierarchical AMOVAs. The significance of AMOVA was tested with 1,000 permutations. Gene flow (*Nm*) among populations for the cpDNA and SSR data was estimated as 1/4(1/*F*_ST_ − 1) (Wright, 1951). Pollen/seed migration ratio (*r*) was estimated using a modified equation of Ennos (1994), according to Petit et al. (2005), *r* = *m*_p_/*m*_s_ = [(1/*F*_ST(n)_ − 1) − 2(1/*F*_ST(c)_ − 1)]/(1/*F*_ST(c)_ − 1). Here, *m*_p_ and *m*_s_ is the pollen migration rate and seed migration ratio, *F*_ST(n)_ and *F*_ST(c)_ is the nuclear (SSR) *F*_ST_ and cytoplasmic (cpDNA) *F*_ST_.

### Species Distribution Models

The potential range shift of *P. stenoptera* was determined by SDM in Maxent version 3.4.1 (Phillips et al., 2006). The geographic coordinates used in SDM analysis were based on a set of 40 presence points (**Table S1**), in which 18 points were adopted from the Chinese Virtual Herbarium, while the other 22 points represent our sampling sites. Nineteen environmental variables at 2.5 arcmin resolution at the present and the last glacial maximum (LGM) were downloaded from the WorldClim website. The calculation method used to determine potential climatically suitable areas was based on the study of Fu et al. (2014). However, logistic probabilities were used for the output. Logistic probabilities higher than 0.5 were taken as high climatically suitable areas in accordance to the classification of Bai et al. (2016). The significant difference of environmental variables among groups was determined by T-test in SPSS version 19 (Inc., Chicago, IL, USA). According to Li et al. (2015), the result of the Qinghai–Tibet movement can be used to represent the difference of warmth and drought between the northern and southern regions of China. Thus, five environmental variables (**Table S2**) related to warmth and drought, including annual mean temperature (Bio1), mean temperature of coldest quarter (Bio11), annual precipitation (Bio12), precipitation of coldest quarter (Bio19), and water vapor pressure in January (wvp1), were selected. The corresponding values, which were downloaded from the WorldClim website, covered the period of 1970–2000 at 2.5 arcmin resolution, then they were further extracted using DIVA-GIS 7.5 (Hijmans et al., 2001).

## Results

### Genetic Diversity

All the four cpDNA noncoding spacers sequenced in *P. stenoptera* showed length variations. The 8 *ndh*Ax1-*ndh*Ax2, 5 *rp*L34-*rp*L36, 9 *trn*L(UAG)-*rp*L32-F, and 11 *trn*V(UAC)x2-*ndh*C haplotype sequences were deposited in GenBank with accession numbers MK002380 to MK002387, MK002389 to MK002393, MK002395 to MK002403, and MK002405 to MK002415, respectively. These sequences, when combined, were aligned with a consensus length of 2,214 bp, and they contained 17 nucleotide substitutions and 15 indels. On the basis of these polymorphisms, 28 haplotypes (H1 to H28) were identified among all the surveyed samples (**Table S3**). With 1–15 mutational steps apart for these haplotypes, H7 and H3 were widespread, and 15 haplotypes were observed in one population only (**Figure 1** and **Table 1**).

**Figure 2:**
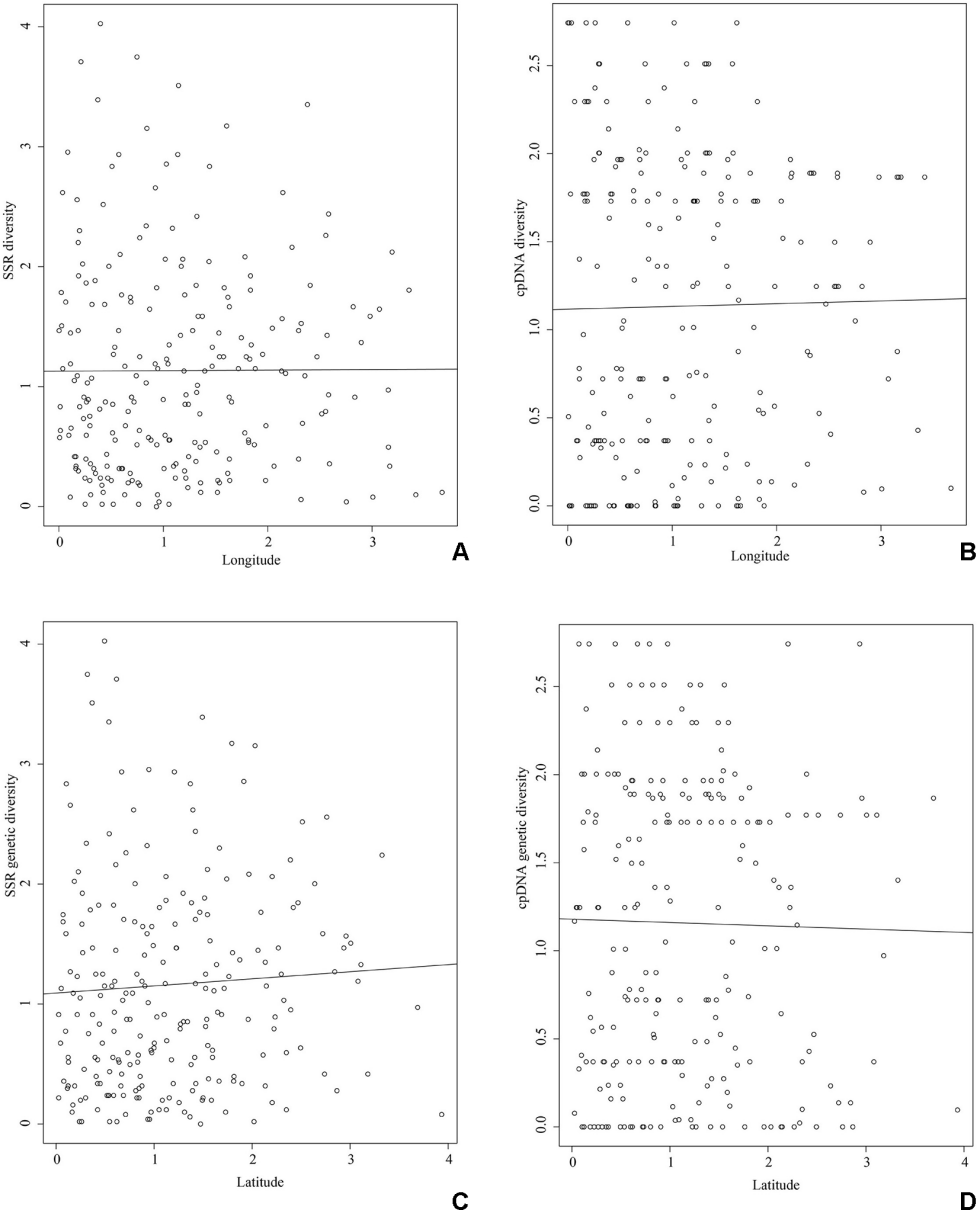
Mantel test between genetic diversity and longitude and latitude. The X axis represents genetic diversity of SSR **(A** and **C)** and cpDNA **(B** and **D)** after standardization. The Y axis represents longitude **(A** and **B)** and latitude **(C** and **D)** after standardization. **(A)** Genetic diversity of SSR along longitude, **(B)** genetic diversity of cpDNA along longitude, **(C)** genetic diversity of SSR along latitude, **(D)** genetic diversity of cpDNA along latitude.

The populations of HBSN and FJWY had the highest nucleotide and haplotype diversities, while the populations of HBSN and HNHM had highest number of private haplotypes (**Table 1**). As for SSR data, 440 individuals of *P. stenoptera* were genotyped. The results of genetic diversity analyses of each population are presented in **Table 1**. The level of genetic diversity (*H*_E_) of each population ranged from 0.194 (JXLH) to 0.397 (HBJG). The level of allelic richness (*A*_R_) of each population ranged from 1.931 (SDTM) to 2.842 (YNYB). Among these populations, six populations were observed with private alleles, and the population of SDMM showed the highest number of private alleles. Mantel test showed that the genetic diversity of SSR (r = 0.004, *P* = 0.390; **Figure 2**) and cpDNA (r = 0.016, *P* = 0.287; **Figure 2**) did not decrease significantly along the longitude direction from west to east. No significant decrease was observed in genetic diversity along the latitude direction (SSR: r = 0.063, *P* = 0.240; cpDNA: r = −0.020, *P* = 0.545; **Figures 2C, D**) from south to north.

### Genetic Structure

**Figure 3:**
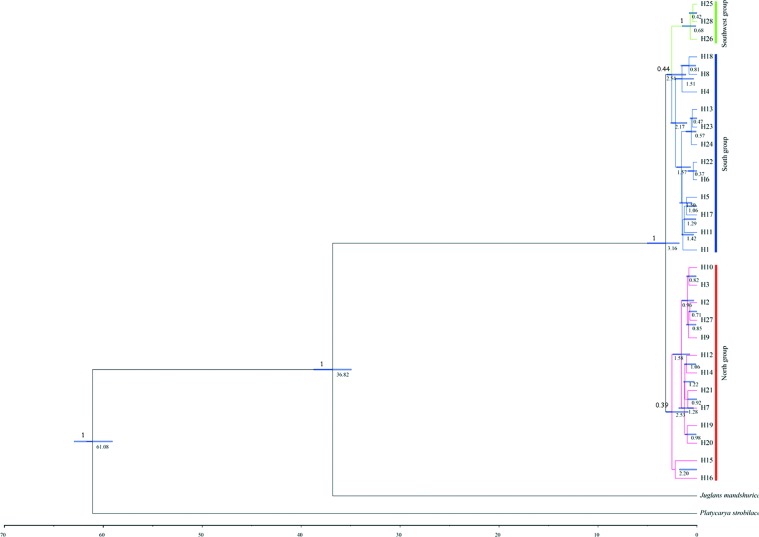
Divergence time of *Pterocarya stenoptera* based on chloroplast DNA (cpDNA) haplotypes estimated with BEAST. Blue bars indicate the 95% credibility intervals of divergence times. The time of divergence of all nodes are showed below branches, and posterior probabilities are given above the main branches.

**Figure 4:**
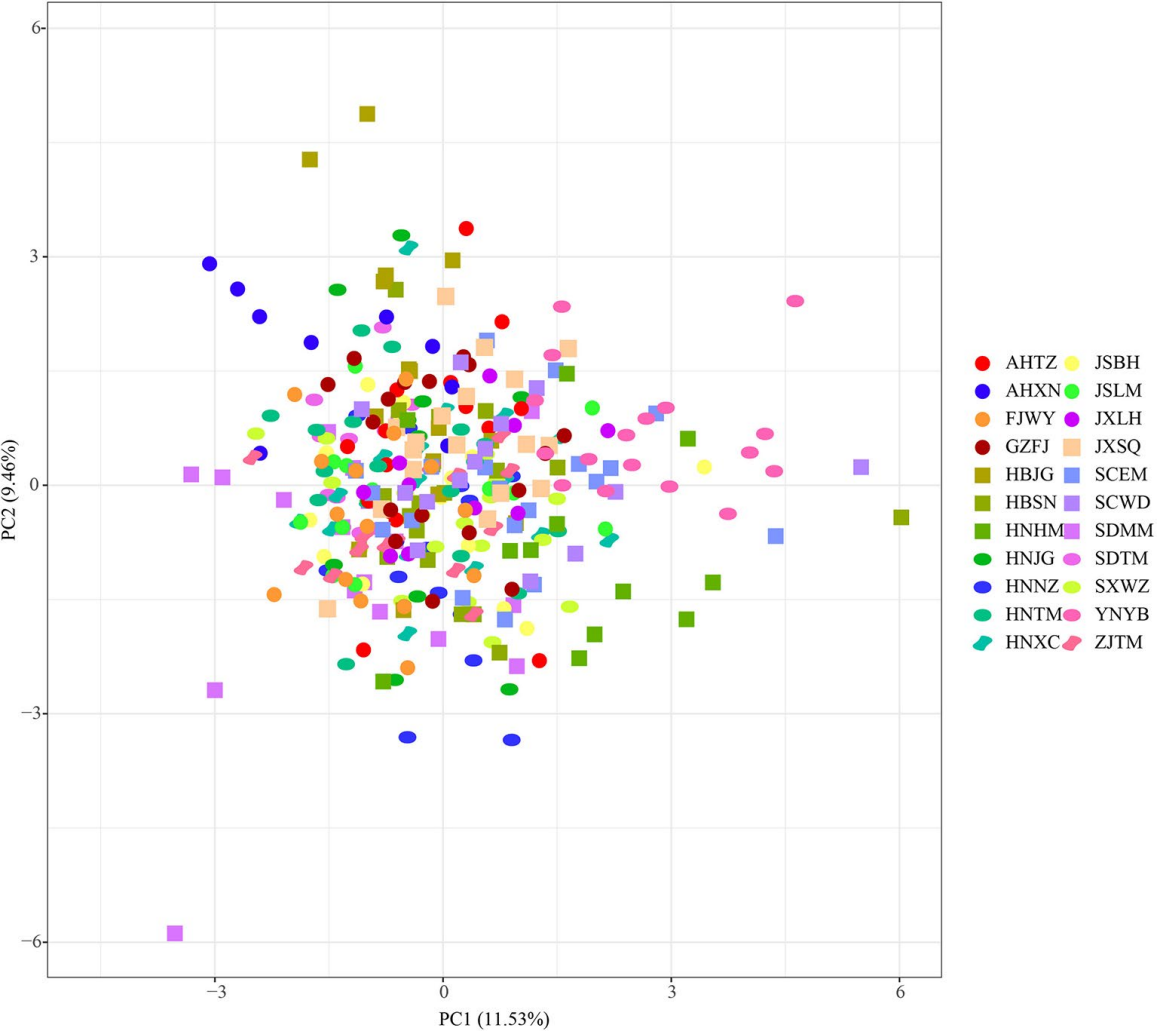
Principal component analysis based on simple sequence repeat (SSR) data. Population codes were showed in **Table 1**.

**Figure 5:**
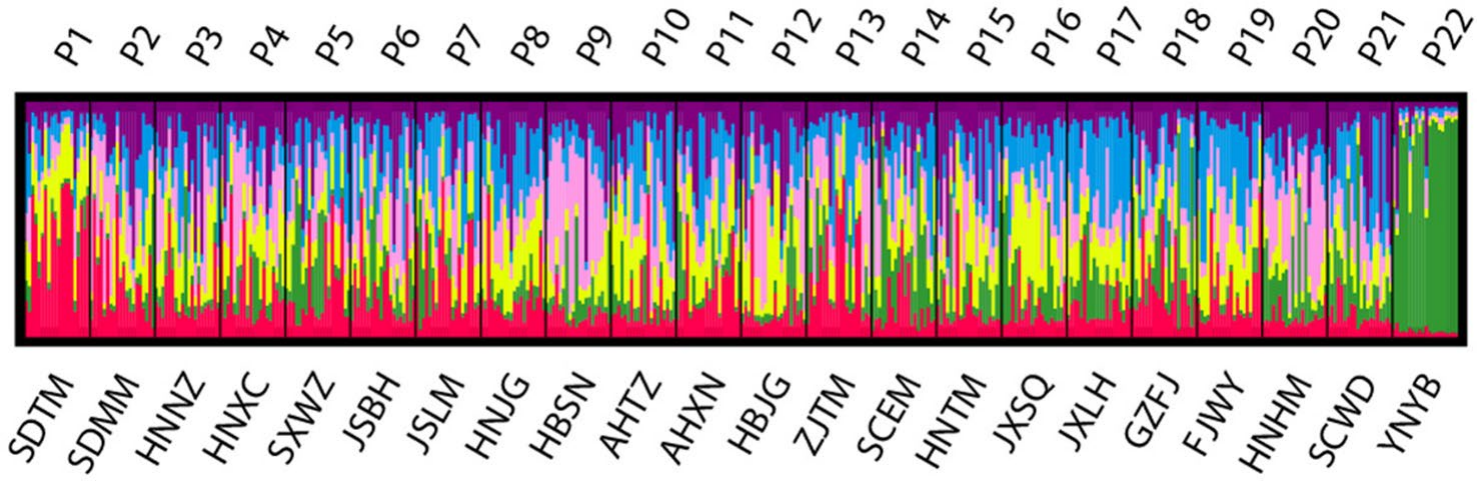
Estimated genetic structure for *K* = 6 obtained with the program STRUCTURE for 22 populations of *Pterocarya stenoptera* based on simple sequence repeat (SSR) data. Each vertical bar represents an individual and its assignment proportion into one of five (colored) population clusters (*K*).

Populations of *P. stenoptera* were significantly structured as revealed by the population-based unweighted pair-group method with arithmetic tree based on cpDNA data, and the 22 populations were subdivided into three genetic groups (**Figure S1**). The three identified genetic groups were the North group, the South group, and the Southwest group. The network of haplotypes also supported this subdivision (**Figures 1** and **3**). These groupings were largely consistent with their geographic distributions. Three haplotypes were shared among groups. In particular, H7 was shared between FJWY from the South group and SDTM, SXWZ, JSBH, JSLM, HNJG, and HBSN from the North group; H17 was shared between ZJTM and HNTM from the South group and the North group; and H23 was shared between SCWD from the Southwest group and SCEM from the South group. According to the Bayesian analysis of population structure, the highest likelihood based on SSR data was obtained when *K* = 6 (**Figure S2**). The best *K* = 1 was estimated according to the value of TI posterior probability (**Figure S3**). The cluster results on TI posterior probability were also supported by principal component analysis. No significant genetic differentiation was found among 22 populations (**Figure 4**). However, the actual results when *K* = 6 in the structure analysis showed that only the population of YNYB can be separated from other populations, whereas the other 21 populations were mixed together and cannot be separated from one another (**Figure 5**). Taken together, the cluster results based on SSR data did not correspond to the separate geographical regions supported by cpDNA data (**Figure S1**). As for cpDNA data, a strong population genetic differentiation (*F*_ST_ = 0.825; *P* < 0.001) was detected by the analyses of molecular variance (AMOVA) (**Table 2**). The relatively low gene flow (*Nm* = 0.053) among populations resulted in significant genetic differentiation. No significant isolations by geographical distance (r = −0.019, *P* = 0.695) and ecological distance (PC1: r = 0.036, *P* = 0.128; PC2: r = −0.055, *P* = 0.946; PC3: r = −0.067, *P* = 0.988; PC4: r = −0.026, *P* = 0.818) for cpDNA were detected at the species-range scale. Nonsignificant isolation by ecological distance for SSR was also detected along PC1, PC3, and PC4 (r = 0.033, *P* = 0.150; r = −0.029, *P* = 0.699; r = −0.027, *P* = 0.791, respectively), but a significant IBE existed along PC2 (r = 0.703, *P* = 0.001). However, significant isolation by geographical distance (r = 0.488, *P* = 0.001) for SSR was detected. AMOVA showed that 54.93% of the total cpDNA genetic differentiation existed among the three groups (*F*_CT_ = 0.549), and 31.50% among populations within groups (*F*_SC_ = 0.699), and 13.58% within populations (*F*_ST_ = 0.864) (**Table 2**). As for SSR data, 2.89% of the total genetic differentiation was found among the three groups (*F*_CT_ = 0.029), 6.34% among populations within groups (*F*_SC_ = 0.065), and 90.78% within populations (*F*_ST_ = 0.092) (**Table 2**). The high pollen/seed migration ratio (*r*) = 52.0 suggests that the high gene flow was mostly transmitted by pollen.

**Table 2:** Nonhierarchical and hierarchical AMOVAs for cpDNA and SSR variation surveyed in populations of *Pterocarya stenoptera* in China.

Source of variation	d.f.	% Total variance	*F*-statistic	*P*-value
*cpDNA*				
Nonhierarchical AMOVAs				
Total	21	82.45%	*F*_ST_ = 0.825	<0.001
North group	11	65.74%	*F*_ST_ = 0.657	<0.001
South group	7	79.12%	*F*_ST_ = 0.791	<0.001
Southwest group	1	51.93%	*F*_ST_ = 0.519	<0.001
Hierarchical AMOVAs				
Among three groups	2	54.93%	*F*_CT_ = 0.549	<0.001
Among populations	19	31.50%	*F*_SC_ = 0.699	<0.001
Within populations	416	13.58%	*F*_ST_ = 0.864	<0.001
*SSR*				
Nonhierarchical AMOVAs				
Total	21	8.13%	*F*_ST_ = 0.081	<0.001
North group	11	5.19%	*F*_ST_ = 0.052	<0.001
South group	7	7.37%	*F*_ST_ = 0.074	<0.001
Southwest group	1	14.98%	*F*_ST_ = 0.150	<0.001
Hierarchical AMOVAs				
Among three groups	2	2.89%	*F*_CT_ = 0.029	<0.05
Among populations	19	6.34%	*F*_SC_ = 0.065	<0.001
Within populations	858	90.78%	*F*_ST_ = 0.092	<0.001

### Divergence Time

Divergence time in the time-calibrated tree (**Figure 3**) ranged from 3.16 Ma (95% HPD = 1.79–5.02) to 0.37 Ma (95% HPD = 0.01–0.88). The divergence time of the North group and the South–Southwest groups was 3.16 Ma (95% HPD = 1.79–5.02), which falls within the A to B stages of the Qinghai–Tibet movement (2.6–3.6 Ma). The divergence time of the South group and the Southwest group was 2.54 Ma (95% HPD = 1.12–3.04), which falls within the B to C stages of the Qinghai–Tibet movement (1.7–2.6 Ma).

### Demographic History

**Figure 6:**
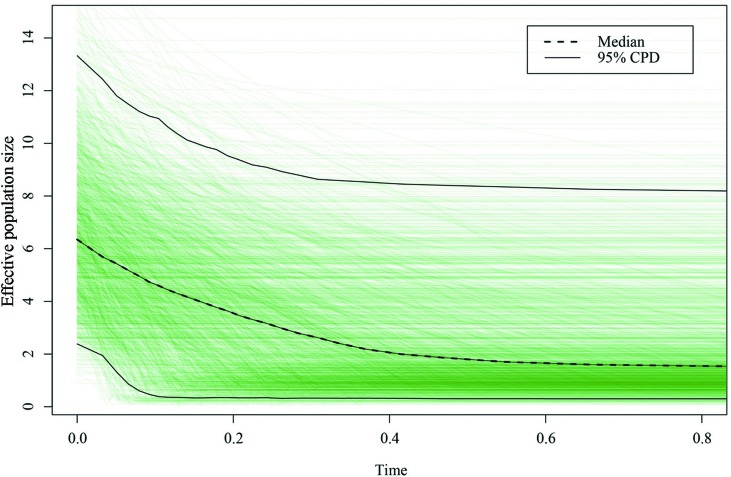
Extended Bayesian skyline analysis based on chloroplast DNA (cpDNA) of *Pterocarya stenoptera.* The x-axis represents the time before present (Ma) and the y-axis represents the effective population size. The dashed and solid lines indicate the median and 95% highest posterior density intervals, respectively.

The neutrality test with Tajima’s *D* and Fu’s *Fs* statistics showed negative values at the total level (Tajima’s *D* = −0.438, *P* = 0.395; Fu’s *Fs* = −3.790, *P* = 0.201; **Table 3**), suggesting that the demographic expansion has occurred in the recent past; however, the obtained values were statistically nonsignificant. This demographic expansion was obviously supported by the nonsignificant sum of squared deviations (SSD) and raggedness (RAG) index values of mismatch distribution analysis at the total level (**Table 3**). Tajima’s *D* and Fu’s *Fs* statistics and mismatch distribution analysis were examined for each of the three groups. The demographic expansion of the South group was supported consistently by the above three methods, whereas the demographic results of the other two groups were inconsistent. EBSP analysis was performed to infer the time of demographic expansion in this study. The results of EBSP indicate a continuous demographic expansion that began approximately 0.40 Ma (**Figure 6**), and this result is highly consistent with the time of Marine Isotope Stage (MIS) 11 (0.33–0.46 Ma).

**Table 3:** Results of demographic analyses based on two datasets for three groups and all samples of *Pterocarya stenoptera*.

Groups	cpDNA			
	SSD (P value)	RAG (P value)	Tajima’s D (P value)	Fu’s FS (P value)
North group	0.026 (0.000)	0.096 (0.000)	−0.406 (0.425)	−3.409 (0.146)
South group	0.005 (0.330)	0.031 (0.560)	−0.175 (0.499)	−0.741 (0.459)
Southwest group	0.014 (0.440)	0.049 (0.760)	−0.850 (0.220)	2.247 (0.875)
Total	0.007 (0.150)	0.023 (0.350)	−0.438 (0.395)	−3.790 (0.201)

SSD, sum-of-squared deviations; Rag, Raggedness index.

## Distribution Inference by Species Distribution Models

**Figure 7:**
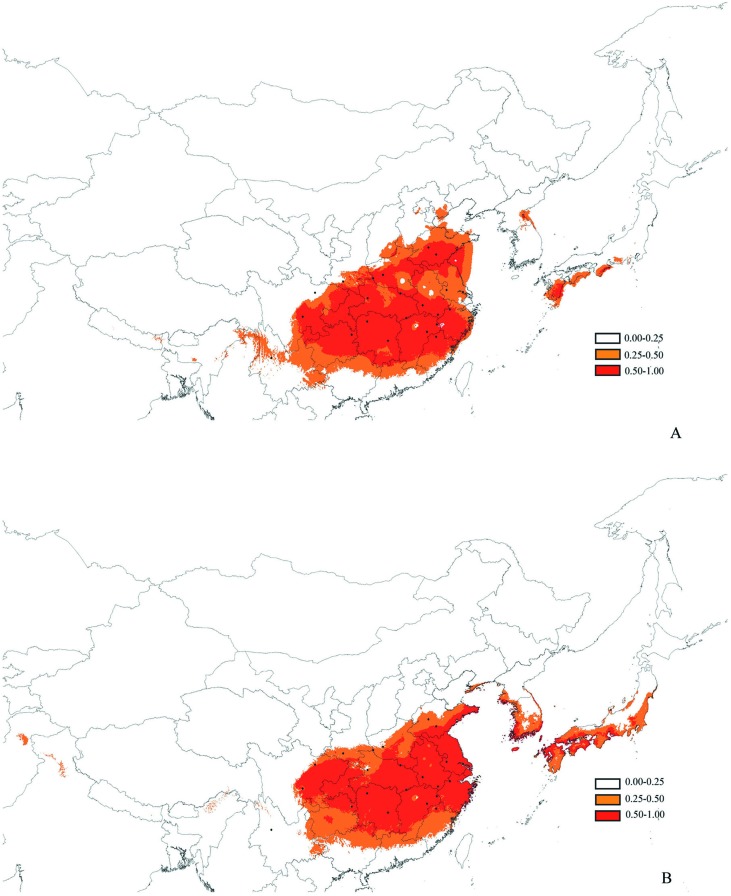
Maps showing potential distribution as a probability of ecological niche modeling with Maxent, distribution maps of *Pterocarya stenoptera* were yielded by software DIVA-GIS. (Light red, logistic value 0–0.25; Dark red, logistic value 0.5–1). **(A)** The Last Glacial Maximum, about 21 thousand years ago, **(B)** the present.

The values of the areas under the receiver operating characteristic curve based on both training and test presence data at the present and lgm periods were 0.994 And 0.990 (Present) 0.994 And 0.992 (Lgm), respectively. The results of areas under the receiver operating characteristic curve values demonstrated good model performance. The results of the predicted distribution of *p. Stenoptera* indicate that the climatically suitable areas (logistic probabilities > 0.25) At present and lgm were both continuous in china, and no habitat fragmentations were isolated by intervening unsuitable habitats (**Figure 7**). The area of climatically suitability (logistic probabilities > 0.25) at the lgm (2,081,410 km^2^) was not compressed compared with that at the present time (1,950,755 km^2^). Similarly, no obvious difference existed between the area of high climatically suitability (logistic probabilities > 0.50) At the lgm (1,221,637 km^2^) and that at the present time (1,172,061 km^2^). The significant difference of the environmental variables (bio1, bio11, bio12, bio19, and wvp1; **Table S2**) among the groups was further determined by t-test in spss. The results showed that the environmental variables, bio1, bio11, bio12, and wvp1 between the north group and the South–southwest groups were significantly different (*p* < 0.05). Moreover, bio12 and bio19 between the South group and the Southwest group were significantly different (*p* < 0.05).

## Discussion

### Spatial Population Genetic Structure of *P. stenoptera*

The spatial genetic structure of species is widely known to be linked to multiple factors (Ohsawa and Ide, 2008; Hickerson et al., 2010; Yang et al., 2017). In this study, we investigated the spatial genetic structure of *P. stenoptera* across its distribution area in China to provide insights into the complex interplay among multiple factors. Our survey showed that the 22 populations of *P. stenoptera* were divided into three groups, i.e., North group, South group, and Southwest group.

Previous phylogeographical studies reported large-scale intraspecific disjunctions in many species that can alternatively be explained by geographical barrier, ecological barrier, and recolonization from multiple refugia (Li et al., 2011; Li et al., 2012; Bai et al., 2016; Luo et al., 2017; Ye et al., 2017). If the subdivision of *P. stenoptera* was caused by geographical barriers among groups, then the premise of this hypothesis was that geographical barriers existed among these groups. For the distribution area of *P. stenoptera*, no large mountains isolated the groups, but three major rivers, i.e., the Yellow River, Huaihe River, and Yangtze River, may have divided them. According to the literature, the Yellow River and Yangtze River, as phylogeographical boundaries, have promoted the genetic divergence of plant species that distributed across these rivers (Wang et al., 2014; Geng et al., 2015). However, the Yangtze River and Yellow River formed at 1.9–1.7 Ma (Wang et al., 1998; Li et al., 2001), while the Huaihe River formed at the end of the Late Pleistocene (ca. 0.15 Ma; Shao et al., 1989), which are clearly later than the divergence time of the North group and the South–Southwest groups (3.16 Ma), and the divergence time of the South group and the Southwest group (2.54 Ma). The findings indicate that these river system formations were not the vicariant factor that have caused the divergence of the three groups. Furthermore, the topologies of these rivers were inconsistent with the geographical boundaries of the three groups (**Figure 1**), indicating that these rivers also did not play a role in enhanced isolation after genetic differentiation. Thus, the divergence of the three groups is unlikely caused by the geographical isolation of *P. stenoptera*. Moreover, the possibility that these larger rivers served as dispersal channels for *P. stenoptera* still needs to be explored. The flow direction of the three large rivers are from west to east, but our study did not find a gradually decline of genetic diversity in this direction (**Figure 2**). Therefore, these rivers did not act as channels of dispersal of *P. stenoptera*.

We used SDM to simulate the distribution of *P. stenoptera* at the present and at LGM to further test whether this divergence was caused by ecological isolation. The results did not show habitat fragmentations isolated by intervening unsuitable habitats at the present and at LGM. Our results of nonsignificant IBE based on cpDNA and SSR data (except PC2) also do not support the assumption that divergence is caused by ecological isolation. Moreover, the Pleistocene glacial epoch occurred after the Kunlun–Yellow River movement (since ca. 1.1 Ma; Li et al., 2015), suggesting a much later than the time of species group differentiation. These findings indicate that the much even earlier glaciations cannot be the reason for the differentiation of these groups. In the assumption that the genetic divergence of *P. stenoptera* is caused by recolonization from multiple refugia, the movement of populations away from a refugia will likely cause a gradual decline in genetic diversity. However, neither the North group nor the South group underwent a gradient descent from the presumed refugia, i.e., these populations have the highest genetic diversity. Here, the Southwest group was not surveyed considering the relatively small number of populations in this area. We also did not find a gradual decline in genetic diversity from south to north. The results of nonsignificant IBD based on cpDNA data also do not support this particular hypothesis. The IBD for SSR data was significant, but they were likely influenced by the strong gene flow *via* pollination. Thus, the results cannot clearly reflect a migration route that occurred in the past. Obviously, our results do not support this interpretation of recolonization from multiple refugia. Taken together, the reasons mentioned above do not seem to explain the genetic differentiation among the three groups.

We then focused on the divergence time between groups. The divergence time of the North group and the South–Southwest group was 3.16 Ma. This divergence time falls within the time of the Qinghai–Tibet movement (1.7–3.6 Ma; Li and Fang, 1998). Consequently, the Qinghai–Tibet movement caused the southeast China, i.e. the South and Southwest groups for *P. stenoptera*, to become more humid until today (Li et al., 2015). Our T-test results also showed that the environmental variables related to temperature, precipitation, and air humidity (Bio1, Bio11, Bio12, and wvp1; **Table S2**) between the North group and the South–Southwest groups were significantly different. The divergence time of the South group and the Southwest group was 2.54 Ma, which falls within the time of the Qinghai–Tibet movement. The Southwest group was closer to Qinghai–Tibet than the South group, and the environmental variables related to precipitation (Bio12 and Bio19) between the South group and the Southwest group were significantly different. The environmental heterogeneity caused by the Qinghai–Tibet movement may be related to the genetic differentiation of the three groups of *P. stenoptera*. Nonetheless, we cannot completely rule out genetic drift and ancient hybridization. Random genetic drift or ancient hybridization to capture the chloroplast genes from other species may also give rise to the result of our study.

For the structure analysis of the SSR data, the results showed that only one population of YNYB can be separated from the other populations, while the other 21 populations were mixed together. This phenomenon is due to the high gene flow among populations (*Nm* = 2.836), which is mainly derived from pollen transmission with the high pollen/seed migration ratio (*r* = 52.0). Efficient gene flow (*Nm* > 1) will break the isolation between populations (Wright, 1931). Although the pollen-mediated gene flow obscured previous genetic structures, the YNYB population at the edge of the distribution area is not mixed with other populations. Efficient gene flow can also explain the access to private haplotypes of only a handful population. However, the eastern marginal population SDMM has an unusual number of private genes, which may be the result of genetic drift.

### Population Dynamics for *P. stenoptera* in Response to Climatic Oscillations During the Pleistocene

Previous phylogeographical studies suggested that the species distributed in China during the Pleistocene glacial period migrated southward or retreated to main refugia as a means to cope with the climate fluctuation (Qiu et al., 2011; Liu et al., 2012). Recent phylogeographical studies offer another alternative hypothesis of microrefugia, i.e., species persisted *in situ* by local adaptation during the multiple glacial–interglacial cycles during the Pleistocene (Fu et al., 2014; Wang et al., 2014; Zhang et al., 2015; Li et al., 2018). In the case of *P. stenoptera*, the genetic differentiation of the three groups with obvious boundaries and the simulation of species distribution for the LGM and present periods obviously did not support the assumption of range shift by southward migration. The results of SDM indicate that the area of climatically suitability at LGM was not compressed compared with that at the present time. Moreover, a gradient decline in genetic diversity was lacking due to the post-glacial colonization of the groups of *P. stenoptera*. Thus, the range in the form of retreat to the main refugia for *P. stenoptera* during the Pleistocene glacial period was not supported. On the basis of the cpDNA data, most of the populations (13 of 22 populations) of *P. stenoptera* have their own private genes. The results indicate the microrefugia hypothesis are likely suitable for *P. stenoptera*. Although our results did not support the range shift of *P. stenoptera* during the climatic fluctuations, its population size was affected. Tajima’s *D* and Fu’s *Fs* statistics and the mismatch distribution analysis all supported *P. stenoptera* experienced a population expansion. The results of EBSP indicate that this expansion occurred at approximately 0.40 Ma whose timeline is highly, consistent with the interglacial of MIS 11 (0.33–0.46 Ma; Cui et al., 2011). In other words, *P. stenoptera* populations experienced continuous population expansion after MIS 12, i.e., the Zhonglianggan glaciation. Overall, the climatic fluctuation in the Pleistocene did not cause the substantial range shift of *P. stenoptera*, but its population size was affected. Therefore, our results support the assumption that *P. stenoptera* has not been compressed into several main refugia during the Pleistocene. The previously proposed microrefugia hypothesis (Miao et al., 2017a) seems to be more suitable for *P. stenoptera*, which underwent local adaptation in response to the climate fluctuations during the Pleistocene.

The main goal of this study was to understand how geological events, climate change, and river systems affect the genetic differentiation and population dynamics of *P. stenoptera*. The phylogeographic study of *P. stenoptera* showed that the species is genetically highly diverse based on the cpDNA data. Our results support the assumption that the environmental heterogeneity, which was caused by the climate change resulting from the Qinghai–Tibet movement may be linked to the genetic divergence. The climatic fluctuations during the Pleistocene did not cause the substantial range shift of *P. stenoptera*, but the fluctuations affected its population size. We also confirm in this study that the river systems in the area did not act as channels or barriers of dispersal for *P. stenoptera*.

## Data Availability Statement

Publicly available datasets were analyzed in this study. This data can be found here: accession numbers MK002380 to MK002387, MK002389 to MK002393, MK002395 to MK002403, and MK002405 to MK002415.

## Author Contributions

YL conceived the research project and wrote the paper. Z-HQ, M-WL, and J-XL collected the data. Z-HQ, Y-XH, M-WL, and X-FY analyzed the data. All authors are in agreement with the content of the manuscript.

## Funding

This work was supported by the National Natural Science Foundation of China (31770225) and Henan Agricultural University Science & Technology Innovation Fund (KJCX2016A2) for population sampling, the Natural Science Foundation of Henan Province (182300410039) for sequencing and genotyping of this study, the Funding Scheme of Young Backbone Teachers of Higher Education Institutions in Henan Province (2015GGJS-081) for all experimental materials of this study.

## Conflict of Interest

The authors declare that the research was conducted in the absence of any commercial or financial relationships that could be construed as a potential conflict of interest.

## References

[B1] An Z. S. Zhang P. Z. Wang E. C. Wang S. M. Qiang X. K. Li L. (2006). Changes of the monsoon-arid environment in China and growth of the Tibetan Plateau since the Miocene. Quat. Sci. 26, 678–693.

[B2] Avise J. C. (2009). Phylogeography: retrospect and prospect. J.Biogeogr. 36, 3–15. 10.1111/j.1365-2699.2008.02032.x

[B3] Bai W. N. Wang W. T. Zhang D. Y. (2016). Phylogeographic breaks within Asian butternuts indicate the existence of a phytogeographic divide in East Asia. New Phytol. 209, 1757–1772. 10.1111/nph.13711 26499508

[B4] Beck M. W. (2017). ggord: Ordination Plots with ggplot2. R package version 1.0.0. https://github.com/fawda123/ggord

[B5] Bouckaert R. Heled J. Kühnert D. Vaughan T. Wu C. H. Xie D. (2014). BEAST 2: a software platform for Bayesian evolutionary analysis. PloS Comput. Biol. 10, e1003537. 10.1371/journal.pcbi.1003537 24722319 PMC3985171

[B6] Caicedo A. L. Schaal B. A. (2004). Population structure and phylogeography of *Solanum pimpinellifolium* inferred from a nuclear gene. Mol. Ecol. 13, 1871–1882. 10.1111/j.1365-294X.2004.02191.x 15189210

[B7] Cazé A. L. R. Mäder G. Nunes T. S. Queiroz L. P. de Oliveira G. Diniz-Filho J. A. F. (2016). Could refuge theory and rivers acting as barriers explain the genetic variability distribution in the Atlantic forest? Mol. Phylogenet. Evol. 101, 242–251. 10.1016/j.ympev.2016.05.013 27188539

[B8] Clement M. Posada D. Crandall K. A. (2000). TCS: a computer program to estimate gene genealogies. Mol. Ecol. 9, 1657–1660. 10.1046/j.1365-294x.2000.01020.x 11050560

[B9] Comes H. P. Kadereit J. W. (1998). The effect of Quaternary climatic changes on plant distribution and evolution. Trends Plant Sci. 3, 432–438. 10.1016/S1360-1385(98)01327-2

[B10] Cui Z. J. Chen Y. X. Zhang W. Zhou S. Z. Zhou L. P. Zhang M. (2011). Research history, glacial chronology and origins of Quaternary glaciations in China. Quat. Sci. 31, 749–764.

[B11] Cui Z. J. Wu Y. Q. Liu G. N. Ge D. K. Pang Q. Q. Xu Q. H. (1998). On Kunlun-Yellow River tectonic movement. Sci. China Ser. D: Earth Sci. 41, 592–600. 10.1007/BF02878741

[B12] Deng T. Ding L. (2015). Paleoaltimetry reconstructions of the Tibetan Plateau: progress and contradictions. Natl. Sci. Rev. 2, 417–437. 10.1093/nsr/nwv062

[B13] Earl D. A. vonHoldt B. M. (2012). STRUCTURE HARVESTER: a website and program for visualizing STRUCTURE output and implementing the Evanno method. Conserv. Genet. Resour. 4, 359–361. 10.1007/s12686-011-9548-7

[B14] Ennos R. A. (1994). Estimating the relative rates of pollen and seed migration among plant populations. Heredity 72, 250–259. 10.1038/hdy.1994.35

[B15] Evanno G. Regnaut S. Goudet J. (2005). Detecting the number of clusters of individuals using the software structure: a simulation study. Mol. Ecol. 14, 2611–2620. 10.1111/j.1365-294X.2005.02553.x 15969739

[B16] Excoffier L. Lischer H. E. L. (2010). Arlequin suite ver 3.5: A new series of programs to perform population genetics analyses under Linux and Windows. Mol. Ecol. Resour. 10, 564–567. 10.1111/j.1755-0998.2010.02847.x 21565059

[B17] Fu Y. X. (1997). Statistical tests of neutrality of mutations against population growth, hitchhiking and background selection. Genetics 147, 915–925. 9335623 10.1093/genetics/147.2.915 PMC1208208

[B18] Fu Z. Z. Li Y. H. Zhang K. M. Li Y. (2014). Molecular data and ecological niche modeling reveal population dynamics of widespread shrub *Forsythia suspensa* (Oleaceae) in China’s warm-temperate zone in response to climate change during the Pleistocene. BMC Evol. Biol. 14, 114. 10.1186/1471-2148-14-114 24885704 PMC4052925

[B19] Geng Q. F. Yao Z. G. Yang J. He J. Wang D. B. Wang Z. S. (2015). Efect of Yangtze River on population genetic structure of the relict plant *Parrotia subaequalis in* eastern China. Ecol. Evol. 5, 4617–4627. 10.1002/ece3.1734 26668727 PMC4670060

[B20] Goudet J. (1995). FSTAT version 2.9.3.2. A computer software to calculate F-statistics. J. Hered. 86, 485–486. 10.1093/oxfordjournals.jhered.a111627

[B21] Hewitt G. M. (2000). The genetic legacy of the Quaternary ice ages. Nature 405, 907–913. 10.1038/35016000 10879524

[B22] Hewitt G. M. (2004). Genetic consequences of climatic oscillations in the Quaternary. Philos. Trans. Roy. Soc Lond. B Biol. Sci. 359, 183–195. 10.1098/rstb.2003.1388 15101575 PMC1693318

[B23] Hickerson M. J. Carstens B. C. Cavendar-Bares J. Crandall K. A. Graham C. H. Johnson J. B. (2010). Phylogeography’s past, present and future: 10 years after Avise, 2000. Mol. Phylogenet. Evol. 54, 291–301. 10.1016/j.ympev.2009.09.016 19755165

[B24] Hijmans R. J. (2015). Geosphere: Spherical Trigonometry. R Package Version 1.5-1. https://rdrr.io/cran/geosphere

[B25] Hijmans R. J. Guarino L. Cruz M. Rojas E. (2001). Computer tools for spatial analysis of plant genetic resources data: 1. DIVA-GIS. Plant Genet. Resour. Newsl. 127, 15–19.

[B26] Jakobsson M. Rosenberg N. A. (2007). CLUMPP: a cluster matching and permutation program for dealing with label switching and multimodality in analysis of population structure. Bioinformatics 23, 1801–1806. 10.1093/bioinformatics/btm233 17485429

[B27] Jiang Z. Y. Wu Y. Q. Cui Z. J. (2005). Kunlun-Yellow River tectonic motion and formation of modern physical geography pattern of China. J. Beijing Norm. Univ. 41, 85–88.

[B28] Kumar S. Stecher G. Li M. Knyaz C. Tamura K. (2018). MEGA X: Molecular Evolutionary Genetics Analysis across computing platforms. Mol. Biol. Evol. 35, 1547–1549. 10.1093/molbev/msy096 29722887 PMC5967553

[B29] Larkin M. A. Blackshields G. Brown N. P. Chenna R. McGettigan P. A. McWilliam H. (2007). Clustal W and Clustal X version 2.0. Bioinformatics 23, 2947–2948. 10.1093/bioinformatics/btm404 17846036

[B30] Li J. J. Fang X. M. Pan B. T. Zhao Z. J. Song Y. G. (2001). Late Cenozoic intensive uplift of Qinghai-Xizang Plateau and its impacts on environments in surrounding area. Quat. Sci. 21, 381–391.

[B31] Li J. J. Fang X. M. (1998). Research on the uplift of the Qinghai Xizang Plateau and environmental changes. Chinese Sci. Bull. 43, 1569–1574.

[B32] Li J. J. Wen S. X. Zhang Q. S. Wang F. B. Zheng B. X. Li B. Y. (1979). A discussion on the period, amplitude and type of the uplift of the Qinghai-Xizang Plateau. Sci. China Ser. A 22, 1314–1328.

[B33] Li J. J. Zhou S. Z. Zhao Z. J. Zhang J. (2015). The Qingzang Movement: The major uplift of the Qinghai-Tibetan Plateau. Sci. China Earth Sci. 58, 2113–2122. 10.1007/s11430-015-5124-4

[B34] Li Y. Yan H. F. Ge X. J. (2012). Phylogeographic analysis and environmental niche modeling of widespread shrub *Rhododendron simsii* in China reveals multiple glacial refugia during the last glacial maximum. J. Syst. Evol. 50, 362–373. 10.1111/j.1759-6831.2012.00209.x

[B35] Li Y. Zhai S. N. Qiu Y. X. Guo Y. P. Ge X. J. Comes H. P. (2011). Glacial survival east and west of the ‘Mekong-Salween-Divide’ in the Himalaya-Hengduan Mountains Region as revealed by AFLP and cpDNA sequence variation in *Sinopodophyllum hexandrum* (Berberidaceae). Mol. Phylogenet. Evol. 59, 412–424. 10.1016/j.ympev.2011.01.009 21296173

[B36] Li Y. Zhang X. X. Mao R. L. Yang J. Miao C. Y. Li Z. (2017). Ten years of landscape genomics: challenges and opportunities. Front. Plant Sci. 8, 2136. 10.3389/fpls.2017.02136 29312391 PMC5733015

[B37] Li Y. C. Zhong D. L. Rao G. Y. Wen J. Ren Y. Zhang J. Q. (2018). Gone with the trees: Phylogeography of *Rhodiola* sect. *Trifida* (Crassulaceae) reveals multiple refugia on the Qinghai-Tibetan Plateau. Mol. Phylogenet. Evol. 121, 110–120. 10.1016/j.ympev.2018.01.001 29309848

[B38] Liang H. Y. Feng Z. P. Pei B. Li Y. Yang X. T. (2018). Demographic expansion of two *Tamarix* species along the Yellow River caused by geological events and climate change in the Pleistocene. Sci. Rep. 8, 60. 10.1038/s41598-017-19034-x 29311687 PMC5758526

[B39] Liu J. Möller M. Provan J. Gao L. M. Poudel R. C. Li D. Z. (2013). Geological and ecological factors drive cryptic speciation of yews in a biodiversity hotspot. New Phytol. 199, 1093–1108. 10.1111/nph.12336 23718262

[B40] Liu J. Q. Sun Y. S. Ge X. J. Gao L. M. Qiu Y. X. (2012). Phylogeographic studies of plants in China: advances in the past and directions in the future. J. Syst. Evol. 50, 267–275. 10.1111/j.1759-6831.2012.00214.x

[B41] Luo D. Xu B. Li Z. M. Sun H. (2017). The ‘Ward Line-Mekong-Salween Divide’ is an important floristic boundary between the eastern Himalaya and Hengduan Mountains: evidence from the phylogeographical structure of subnival herbs *Marmoritis complanatum* (Lamiaceae). Bot. J. Linn. Soc 185, 482–496. 10.1093/botlinnean/box067

[B42] Miao C. Y. Yang J. Mao R. L. Li Y. (2017a). Phylogeography of *Achyranthes bidentata* (Amaranthaceae) in China’s warm-temperate zone inferred from chloroplast and nuclear DNA: insights into population dynamics in response to climate change during the Pleistocene. Plant Mol. Biol. Rep. 35, 166–176. 10.1007/s11105-016-1013-z

[B43] Miao C. Y. Li Y. Yang J. Mao R. L. (2017b). Landscape genomics reveal that ecological character determines adaptation: a case study in smoke tree (*Cotinus coggygria* Scop.). BMC Evol. Biol. 17, 202. 10.1186/s12862-017-1055-3 28835216 PMC5569454

[B44] Nei M. (1987). Molecular evolutionary genetics. New York: Columbia University Press. 10.7312/nei-92038

[B45] Ohsawa T. Ide Y. (2008). Global patterns of genetic variation in plant species along vertical and horizontal gradients on mountains. Global Ecol. Biogeogr. 17, 152–163. 10.1111/j.1466-8238.2007.00357.x

[B46] Oksanen J. Blanchet F. G. Friendly M. Kindt R. Legendre P. McGlinn D. (2017). Vegan: Community Ecology Package. R Package Version 2.4-5. https://github.com/vegandevs/vegan

[B47] Petit R. J. Aguinagalde I. de Beaulieu J. L. Bittkau C. Brewer S. Cheddadi R. (2003). Glacial refugia: hotspots but not melting pots of genetic diversity. Science 300, 1563–1565. 10.1126/science.1083264 12791991

[B48] Petit R. J. Duminil J. Fineschi S. Salvini D. Vendramin G. G. (2005). Comparative organization of chloroplast, mitochondrial and nuclear diversity in plant populations. Mol. Ecol. 14, 689–701. 10.1111/j.1365-294X.2004.02410.x 15723661

[B49] Phillips S. J. Anderson R. P. Schapire R. E. (2006). Maximum entropy modeling of species geographic distributions. Ecol. Model. 190, 231–259. 10.1016/j.ecolmodel.2005.03.026

[B50] Pluess A. R. Frank A. Heiri C. Lalagüe H. Vendramin G. G. Oddou-Muratorio S. (2016). Genome-environment association study suggests local adaptation to climate at the regional scale in *Fagus sylvatica* . New Phytol. 210, 589–601. 10.1111/nph.13809 26777878

[B51] Pritchard J. K. Stephens M. Donnelly P. (2000). Inference of population structure using multilocus genotype data. Genetics 155, 945–959. 10835412 10.1093/genetics/155.2.945 PMC1461096

[B52] Qiu Y. X. Fu C. X. Comes H. P. (2011). Plant molecular phylogeography in China and adjacent regions: Tracing the genetic imprints of Quaternary climate and environmental change in the world’s most diverse temperate flora. Mol. Phylogenet. Evol. 59, 225–244. 10.1016/j.ympev.2011.01.012 21292014

[B53] Rambaut A. (2016). FigTree, version 1.4.3. Computer Program Distributed by the Author. Edinburgh U. K. http://tree.bio.ed.ac.uk/software/figtree/

[B54] Rea D. K. Snoeckx H. Joseph L. H. (1998). Late Cenozoic eolian deposition in the North Pacific: Asian drying, Tibetan uplift, and cooling of the northern hemisphere. Paleoceanography 13, 215–224. 10.1029/98PA00123

[B55] Rosenberg N. A. (2004). DISTRUCT: a program for the graphical display of population structure. Mol. Ecol. Notes 4, 137–138. 10.1046/j.1471-8286.2003.00566.x

[B56] Rozas J. Ferrer-Mata A. Sánchez-DelBarrio J. C. Guirao-Rico S. Librado P. Ramos-Onsins S. E. (2017). DnaSP 6: DNA sequence polymorphism analysis of large data sets. Mol. Biol. Evol. 34, 3299–3302. 10.1093/molbev/msx248 29029172

[B57] Shao S. X. Guo S. Q. Han S. H. (1989). Geomorphic structures and evolution of Huang-Huai Plain in china. Acta Geogr. Sin. 44, 314–322. 10.11821/xb198903007

[B58] Shaw J. Lickey E. B. Schilling E. E. Small R. L. (2007). Comparison of whole chloroplast genome sequences to choose noncoding regions for phylogenetic studies in Angiosperms: the tortoise and the hare III. Am. J. Bot. 94, 275–288. 10.3732/ajb.94.3.275 21636401

[B59] Shi Y. F. (1998). Evolution of the cryosphere in the Tibetan Plateau, China, and its relationship with the global change in the Mid Quaternary. J. Glaciol. Geocryol. 20, 197–208. 10.1088/0256-307X/16/9/027

[B60] Shi Y. F. Cui Z. J. Su Z. (2006). The Quaternary glaciations and enviromental changes in China. Shijiazhuang: Hebei Science and Technology Publishing Press.

[B61] Tajima F. (1989). Statistical method for testing the neutral mutation hypothesis by DNA polymorphism. Genetics 123, 585–595. 2513255 10.1093/genetics/123.3.585 PMC1203831

[B62] Tamura K. Nei M. Kumar S. (2004). Prospects for inferring very large phylogenies by using the neighbor-joining method. Proc. Natl. Acad. Sci. U. S. A. 101, 11030–11035. 10.1073/pnas.0404206101 15258291 PMC491989

[B63] Verity R. Nichols R. A. (2016). Estimating the number of subpopulations (K) in structured populations. Genetics 203, 1827–1839. 10.1534/genetics.115.180992 27317680 PMC4981280

[B64] Wang F. B. Li S. F. Shen X. H. (1998). “Geological Record From Outcrops in Northern Slope of the Middle Section of the Himalaya (Mainly the Gyirong Basin),” in Late Cenozoic Uplift of Tibet and Environment Changes. Eds. Shi Y. F. Li J. J. Li B. Y. (Guangzhou: Guangdong Science & Technology Press), 117–139.

[B65] Wang P. F. Li Y. Qian Z. H. Li J. X. Ge X. J. (2018). Isolation and characterization of microsatellite loci from *Pterocarya stenoptera* (Juglandaceae). Appl. Plant Sci. 6, e1205. 10.1002/aps3.1205 PMC6303151 30598863

[B66] Wang W. Tian C. Y. Li Y. H. Li Y. (2014). Molecular data and ecological niche modelling reveal the phylogeographic pattern of *Cotinus coggygria* (Anacardiaceae) in China’s warm-temperate zone. Plant Biol. 16, 1114–1120. 10.1111/plb.12157 24494998

[B67] Wright S. (1931). Evolution in Mendelian populations. Genetics 16, 97–159. 17246615 10.1093/genetics/16.2.97 PMC1201091

[B68] Wright S. (1951). The genetical structure of populations. Ann. Hum. Genet. 166, 323–354. 10.1111/j.1469-1809.1949.tb02451.x 24540312

[B69] Wu Z. Y. (1987). “Origin and Evolution of flora of Xizang,” in Flora xizangica, vol. 5 . Ed. Wu Z. Y. (Beijing: Science Press), 874–902.

[B70] Xu Y. M. Zhou M. H. Shi Y. H. Hu X. Y. Yuan K. K. (2002). Advance on the biological properties and resources utilization of *Pterocarya stenoptera*. J. Northeast Fore. Univ. 30, 42–48. 10.1007/s11769-002-0026-8

[B71] Yang J. Miao C. Y. Mao R. L. Li Y. (2017). Landscape population genomics of forsythia (*Forsythia suspensa*) reveal that ecological habitats determine the adaptive evolution of species. Front. Plant Sci. 8, 481. 10.3389/fpls.2017.00481 28424728 PMC5380681

[B72] Ye J. W. Bai W. N. Bao L. Wang T. M. Wang H. F. Ge J. P. (2017). Sharp genetic discontinuity in the aridity-sensitive *Lindera obtusiloba* (Lauraceae): solid evidence supporting the Tertiary floral subdivision in East Asia. J. Biogeogr. 44, 2082–2095. 10.1111/jbi.13020

[B73] Zhang J. B. Li R. Q. Xiang X. G. Manchester S. R. Lin L. Wang W. (2013). Integrated fossil and molecular data reveal the biogeographic diversification of the eastern Asian-eastern North American disjunct hickory genus (*Carya* Nutt.). PloS One 8, e70449. 10.1371/journal.pone.0070449 23875028 PMC3713062

[B74] Zhang T. C. Comes H. P. Sun H. (2011). Chloroplast phylogeography of *Terminalia franchetii* (Combretaceae) from the eastern SinoHimalayan region and its correlation with historical river capture events. Mol. Phylogenet. Evol. 60, 1–12. 10.1016/j.ympev.2011.04.009 21545839

[B75] Zhang X. W. Li Y. Liu C. Y. Xia T. Zhang Q. Fang Y. M. (2015). Phylogeography of the temperate tree species *Quercus acutissima* in China: Inferences from chloroplast DNA variations. Biochem. Syst. Ecol. 63, 190–197. 10.1016/j.bse.2015.10.010

[B76] Zhou S. Z. Wang X. Wang J. Xu L. B. (2006). A preliminary on timing of the oldest Pleistocene glaciation in Qinghai-Xizang Plateau. Quat. Sci. 154–155, 44–51. 10.1016/j.quaint.2006.02.002

