# Supplementary material for: Molecular Phylogeography Analysis Reveals Population Dynamics and Genetic Divergence of a Widespread Tree Pterocarya stenoptera in China
Source: Front Genet. 2019 Nov 1;10:1089. doi: 10.3389/fgene.2019.01089 (PMC6838215; doi:10.3389/fgene.2019.01089)
Supplement: Supplementary file 1 [file DataSheet_1.doc]

Supplementary Material

**Molecular phylogeography analysis reveals population dynamics and genetic divergence of a widespread tree *Pterocarya stenoptera* in China**

**Zhi-Hao Qian^1^, Yong Li^1^*，Ming-Wan Li^1^, Yan-Xia He^2^, Jia-Xin Li^1^, Xiao-Fan Ye^1^**

*** Correspondence:** Yong Li: liyongrui1@126.com

# 1 Supplementary Tables

**Table S1 | Geographic coordinates of *Pterocarya stenoptera* in the analysis of species distribution modeling.**

| **Sample No.** | **Sample location** | **longitude** | **latitude** |
| --- | --- | --- | --- |
| 1 | Tai Mt., Shandong | 117.12 | 36.22 |
| 2 | Meng Mt., Shandong | 117.96 | 35.56 |
| 3 | Nanzhao, Henan | 112.18 | 33.59 |
| 4 | Xichuan, Henan | 111.12 | 33.28 |
| 5 | Wuzi Mt., Shaanxi | 107.84 | 32.95 |
| 6 | Baohua Mt., Jiangshu | 119.09 | 32.14 |
| 7 | Lang Mt., Jiangshu | 120.89 | 31.95 |
| 8 | Jigong Mt., Henan | 114.08 | 31.81 |
| 9 | Shengnongjia, Hubei | 110.5 | 31.37 |
| 10 | Tianzhu Mt., Anhui | 116.49 | 30.67 |
| 11 | Xiuning, Anhui | 118.17 | 29.78 |
| 12 | Jiugong Mt., Hubei | 114.71 | 29.45 |
| 13 | Tianmu Mt., Zhejiang | 119.46 | 30.28 |
| 14 | Emei Mt., Sichuan | 103.44 | 29.57 |
| 15 | Tianmeng Mt., Hunan | 110.46 | 29.11 |
| 16 | Shanqing Mt., Jiangxi | 118.04 | 28.84 |
| 17 | Longhu Mt., Jiangxi | 116.97 | 28.12 |
| 18 | Fengjing Mt., Guizhou | 108.77 | 27.84 |
| 19 | Wuyi Mt., Fujian | 117.97 | 27.65 |
| 20 | Heng Mt., Hunan | 112.72 | 27.26 |
| 21 | Wuduzhen, Sichuan | 104.78 | 31.88 |
| 22 | Yangbi, Yunnan | 100.03 | 25.62 |
| 23 | Kunyu Mt., Shandong | 121.78 | 37.23 |
| 24 | Lao Mt., Shandong | 120.46 | 36.15 |
| 25 | Tongbai Mt., Henan | 113.29 | 32.42 |
| 26 | Baiyun Mt., Henan | 111.86 | 33.68 |
| 27 | Xinghua Mt., Henan | 112.28 | 33.14 |
| 28 | Nangong Mt., Shaanxi | 109.02 | 32.23 |
| 29 | Zijing Mt., Jiangshu | 118.85 | 32.07 |
| 30 | Santan, Hubei | 113.94 | 31.86 |
| 31 | Yandang Mt., Zhejiang | 121.07 | 28.37 |
| 32 | Tiantai Mt., Zhejiang | 121.04 | 29.17 |
| 33 | Lingyan Mt., Sichuan | 103.62 | 31.01 |
| 34 | Yangming Mt., Hunan | 111.93 | 26.08 |
| 35 | Yizhang, Hunan | 112.97 | 25.23 |
| 36 | Yuelu Mt., Hunan | 112.94 | 28.19 |
| 37 | Yi Mt., Jiangxi | 115.04 | 29.37 |
| 38 | Meiling, Jiangxi | 115.73 | 28.78 |
| 39 | Wangmo,Guizhou | 106.10 | 25.17 |
| 40 | Yimen, Yunnan | 102.26 | 24.90 |

**Table S2 | Environmental variables for each location from the WorldClim database.**

|  | **Bio1** | **Bio11** | **Bio12** | **Bio19** | **Wvp1** |
| --- | --- | --- | --- | --- | --- |
| 1.SDTM | 11.789 | -1.188 | 749 | 23 | 0.240 |
| 2.SDMM | 12.676 | -0.538 | 763 | 30 | 0.290 |
| 3.HNNZ | 14.005 | 2.679 | 805 | 37 | 0.339 |
| 4.HNXC | 15.168 | 3.866 | 839 | 37 | 0.403 |
| 5.SXWZ | 13.956 | 3.473 | 876 | 31 | 0.474 |
| 6.JSBH | 15.237 | 3.654 | 1027 | 100 | 0.514 |
| 7.JSLM | 15.433 | 4.332 | 1021 | 114 | 0.553 |
| 8.HNJG | 14.830 | 3.519 | 1141 | 91 | 0.463 |
| 9.HBSN | 11.925 | 2.281 | 1204 | 64 | 0.479 |
| 10.AHTZ | 16.027 | 5.143 | 1502 | 136 | 0.550 |
| 11.AHXN | 16.523 | 5.361 | 1607 | 201 | 0.639 |
| 12.HBJG | 15.705 | 5.139 | 1543 | 176 | 0.537 |
| 13.ZJTM | 15.347 | 4.291 | 1383 | 167 | 0.586 |
| 14.SCEM | 16.319 | 7.025 | 1515 | 60 | 0.633 |
| 15.HNTM | 16.823 | 6.471 | 1391 | 110 | 0.638 |
| 16.JXSQ | 16.698 | 6.258 | 1787 | 236 | 0.649 |
| 17.JXLH | 18.244 | 7.711 | 1783 | 242 | 0.732 |
| 18.GZFJ | 15.146 | 5.594 | 1222 | 89 | 0.626 |
| 19.FJWY | 18.411 | 8.783 | 1744 | 226 | 0.757 |
| 20.HNHM | 17.120 | 6.567 | 1517 | 216 | 0.713 |
| 21.SCWD | 15.702 | 6.142 | 916 | 18 | 0.625 |
| 22.YNYB | 15.048 | 8.822 | 887 | 67 | 0.651 |

Table S3 | Chloroplast DNA sequence polymorphisms detected in four intergenic spacer (IGS) regions of *Pterocarya stenoptera* identifying twenty-eight haplotypes (H1–H28). All sequences are relative to the reference haplotype H1. Numbers 1/0 in sequences denote presence/absence of length polymorphism, identified by superscript letter (a, b, c, d, e, f, g, h, i, j, k, l, m, n, o, p).

| **Haplotype** | **Nucleotide position** | | | | | | | | | | | | | | | | | | | | | | | | | | | | | | | |
| --- | --- | --- | --- | --- | --- | --- | --- | --- | --- | --- | --- | --- | --- | --- | --- | --- | --- | --- | --- | --- | --- | --- | --- | --- | --- | --- | --- | --- | --- | --- | --- | --- |
|  | **ndhAx1-ndhAx2** | | | | | | | **rpL34-rpL36** | | | | **trnL(UAG)-rpL32-F** | | | | | | | | | | | | **trnV(UAC)x2-ndhC** | | | | | | | | |
|  |  |  |  |  |  |  |  |  |  |  |  | 1 | 1 | 1 | 1 | 1 | 1 | 1 | 1 | 1 | 1 | 1 | 1 | 1 | 1 | 1 | 2 | 2 | 2 | 2 | 2 | 2 |
|  |  |  | 2 | 3 | 3 | 4 | 4 | 7 | 7 | 8 | 9 | 0 | 0 | 0 | 2 | 2 | 4 | 5 | 5 | 5 | 5 | 6 | 6 | 8 | 9 | 9 | 0 | 0 | 0 | 1 | 1 | 1 |
|  | 7 | 9 | 3 | 6 | 8 | 2 | 6 | 1 | 8 | 0 | 2 | 5 | 9 | 9 | 2 | 5 | 8 | 1 | 3 | 5 | 9 | 5 | 9 | 3 | 3 | 8 | 4 | 5 | 6 | 0 | 2 | 5 |
|  | 3 | 9 | 6 | 8 | 9 | 3 | 9 | 3 | 8 | 7 | 3 | 7 | 2 | 8 | 9 | 0 | 8 | 5 | 4 | 4 | 9 | 3 | 3 | 4 | 6 | 0 | 5 | 0 | 7 | 1 | 4 | 0 |
| H1 | T | C | 0 | A | C | 0 | 0 | 1^d^ | A | 0 | 1^g^ | 0 | 0 | 0 | C | T | T | T | T | A | C | 1^k^ | G | 1^l^ | G | 1^m^ | C | T | A | 0 | 0 | 1^p^ |
| H2 | T | C | 0 | A | C | 0 | 0 | 1^d^ | A | 0 | 1^g^ | 0 | 0 | 0 | C | T | T | T | T | A | C | 1^k^ | G | 1^l^ | G | 1^m^ | C | T | A | 1^n^ | 0 | 0 |
| H3 | T | C | 0 | A | C | 0 | 0 | 1^d^ | A | 1^f^ | 1^g^ | 0 | 0 | 0 | C | T | T | T | T | A | C | 1^k^ | G | 1^l^ | G | 0 | C | T | A | 1^n^ | 0 | 0 |
| H4 | T | C | 0 | A | C | 0 | 0 | 1^d^ | A | 1^f^ | 1^g^ | 0 | 0 | 0 | C | T | T | T | T | A | C | 1^k^ | G | 1^l^ | G | 1^m^ | C | T | T | 0 | 0 | 0 |
| H5 | T | C | 0 | A | C | 0 | 0 | 1^d^ | A | 1^f^ | 1^g^ | 0 | 0 | 0 | C | T | T | G | T | A | C | 1^k^ | G | 1^l^ | G | 1^m^ | C | T | A | 0 | 0 | 0 |
| H6 | T | C | 1^a^ | A | C | 0 | 0 | 1^d^ | A | 1^f^ | 1^g^ | 0 | 0 | 0 | C | T | T | T | T | A | C | 1^k^ | G | T | G | 1^m^ | C | T | A | 0 | 0 | 0 |
| H7 | T | C | 0 | A | C | 0 | 0 | 1^d^ | A | 1^f^ | 1^g^ | 0 | 0 | 0 | C | T | T | T | T | A | C | 1^k^ | G | 1^l^ | G | 1^m^ | C | T | T | 1^n^ | 0 | 0 |
| H8 | T | C | 0 | A | C | 0 | 0 | 1^d^ | C | 1^f^ | 1^g^ | 0 | 0 | 0 | C | T | T | T | T | A | C | 1^k^ | G | 1^l^ | G | 1^m^ | C | T | T | 0 | 0 | 0 |
| H9 | T | C | 0 | A | C | 0 | 0 | 1^d^ | A | 1^f^ | 1^g^ | 0 | 1^i^ | 0 | C | T | T | T | T | A | C | 1^k^ | G | 1^l^ | G | 1^m^ | C | T | A | 1^n^ | 0 | 0 |
| H10 | T | C | 0 | A | C | 0 | 0 | 1^d^ | A | 1^f^ | 1^g^ | 1^h^ | 0 | 0 | T | T | T | T | T | A | C | 1^k^ | G | 1^l^ | G | 1^m^ | C | T | A | 1^n^ | 0 | 0 |
| H11 | T | C | 0 | A | C | 0 | 0 | 1^e^ | A | 1^f^ | 1^g^ | 0 | 0 | 0 | C | T | T | T | T | A | C | 1^k^ | G | 1^l^ | G | 1^m^ | C | T | A | 0 | 0 | 0 |
| H12 | T | G | 0 | A | C | 1^b^ | 0 | 1^d^ | A | 1^f^ | 1^g^ | 0 | 0 | 0 | C | T | T | T | T | A | C | 1^k^ | G | 1^l^ | G | 1^m^ | C | T | T | 1^n^ | 0 | 0 |
| H13 | T | G | 0 | A | C | 1^b^ | 0 | 1^d^ | A | 1^f^ | 1^g^ | 0 | 0 | 0 | C | T | T | T | T | A | C | 1^k^ | G | 1^l^ | G | 1^m^ | C | T | A | 0 | 0 | 0 |
| H14 | A | C | 0 | A | C | 0 | 0 | 1^d^ | A | 1^f^ | 1^g^ | 0 | 0 | 0 | C | T | T | T | T | A | C | 1^k^ | G | 1^l^ | G | 1^m^ | C | T | T | 1^n^ | 0 | 0 |
| H15 | T | C | 0 | A | C | 0 | 0 | 1^d^ | A | 1^f^ | 1^g^ | 0 | 0 | 0 | C | T | T | T | T | A | C | 1^k^ | G | 1^l^ | G | 1^m^ | C | T | A | 1^n^ | 1^o^ | 0 |
| H16 | T | C | 0 | A | C | 0 | 0 | 1^d^ | A | 1^f^ | 1^g^ | 0 | 0 | 0 | C | T | T | T | T | A | C | 1^k^ | G | 1^l^ | G | 1^m^ | C | T | T | 1^n^ | 1^o^ | 0 |
| H17 | T | C | 0 | A | C | 0 | 0 | 1^d^ | A | 1^f^ | 1^g^ | 0 | 0 | 0 | C | T | T | T | T | A | C | 1^k^ | G | 1^l^ | G | 1^m^ | C | T | A | 0 | 0 | 0 |
| H18 | T | C | 0 | A | C | 0 | 0 | 1^d^ | C | 1^f^ | 1^g^ | 0 | 0 | 1^j^ | C | T | T | T | T | A | A | 1^k^ | A | 1^l^ | G | 1^m^ | C | T | T | 0 | 0 | 0 |
| H19 | T | C | 0 | A | C | 0 | 0 | 1^d^ | A | 1^f^ | 1^g^ | 0 | 0 | 0 | C | T | T | T | T | A | C | 1^k^ | G | 1^l^ | A | 1^m^ | C | T | T | 1^n^ | 0 | 0 |
| H20 | T | C | 0 | A | C | 0 | 0 | 1^d^ | A | 1^f^ | 1^g^ | 0 | 0 | 0 | C | T | T | T | G | A | C | 1^k^ | G | 1^l^ | G | 1^m^ | C | T | T | 1^n^ | 0 | 0 |
| H21 | T | C | 0 | A | C | 0 | 0 | 1^d^ | A | 1^f^ | 1^g^ | 0 | 0 | 0 | C | G | T | T | T | A | C | 1^k^ | G | 1^l^ | G | 1^m^ | C | T | T | 1^n^ | 0 | 0 |
| H22 | T | C | 1^a^ | A | C | 0 | 0 | 1^d^ | A | 1^f^ | 1^g^ | 0 | 0 | 0 | C | T | T | T | T | G | C | 1^k^ | G | 1^l^ | G | 1^m^ | C | T | A | 0 | 0 | 0 |
| H23 | T | C | 0 | A | C | 1^b^ | 0 | 1^d^ | A | 1^f^ | 1^g^ | 0 | 0 | 0 | C | T | T | T | T | A | C | 1^k^ | G | 1^l^ | G | 1^m^ | C | T | A | 0 | 0 | 0 |
| H24 | T | C | 0 | C | C | 1^b^ | 0 | 1^d^ | A | 1^f^ | 1^g^ | 0 | 0 | 0 | C | T | T | T | T | A | C | 1^k^ | G | 1^l^ | G | 1^m^ | C | T | A | 0 | 0 | 0 |
| H25 | T | C | 0 | A | A | 0 | 0 | 1^d^ | A | 1^f^ | 1^g^ | 0 | 0 | 0 | C | T | G | T | T | A | C | 0 | G | 0 | G | 1^m^ | A | C | A | 0 | 0 | 0 |
| H26 | T | C | 0 | A | A | 0 | 1^c^ | 1^d^ | A | 1^f^ | 1^g^ | 0 | 0 | 0 | C | T | G | T | T | A | C | 0 | G | 0 | G | 1^m^ | A | C | T | 0 | 0 | 0 |
| H27 | T | C | 0 | A | C | 0 | 0 | 1^d^ | A | 1^f^ | 1^g^ | 0 | 0 | 0 | C | T | T | T | T | A | C | 1^k^ | G | 1^l^ | G | 1^m^ | C | T | A | 1^n^ | 0 | 0 |
| H28 | T | C | 0 | A | A | 0 | 0 | 1^d^ | A | 1^f^ | 0 | 0 | 0 | 0 | C | T | G | T | T | A | C | 0 | G | 0 | G | 1^m^ | A | C | A | 0 | 0 | 0 |
| a, ATAACAA; b, TTATATTA; c, TATCTAATTTAATTCATTAATCCGAATAAT; d, TTGTATCTC; e, GAGATACAA; f, GTTGAATCA; g, AATCTTAAG; h, TATAT; i, TATAAAT; j, TATAAAA; k, GTACT; l, TAGAA; m, TATAAAGAACT; n, TATTATTATAAATTTTTATTATT; o, TATTATTTATTATTA; p, ATTTTTATTATTTATTATTATAA. | | | | | | | | | | | | | | | | | | | | | | | | | | | | | | | | |


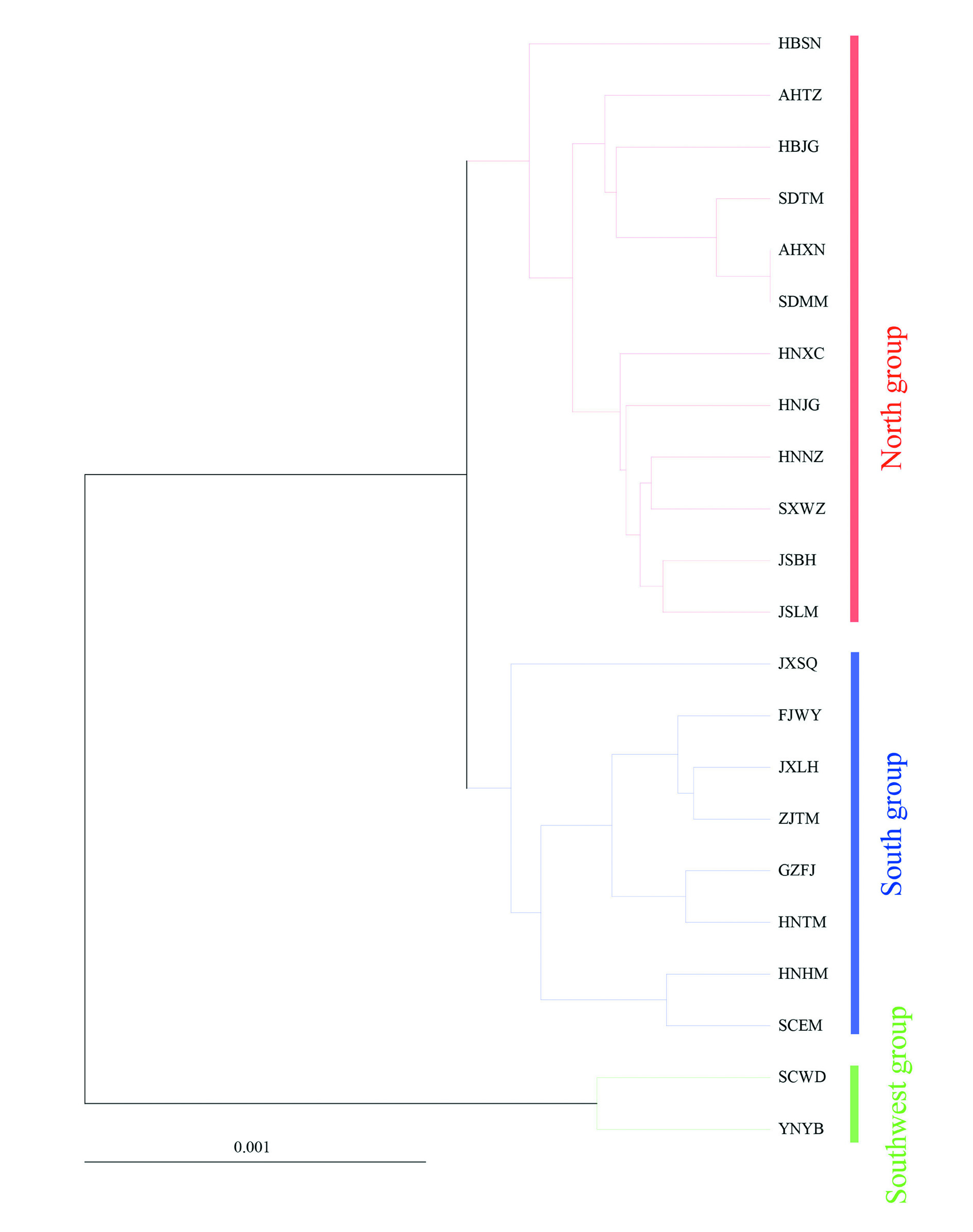


**Figure s1 | Neighbor-joining clustering of 22 populations of *Pterocarya stenoptera* based on their pairwise genetic distances in cpDNA as determined net average between populations of sequences (*D*_A_)**. Population codes are identified in Table 1.


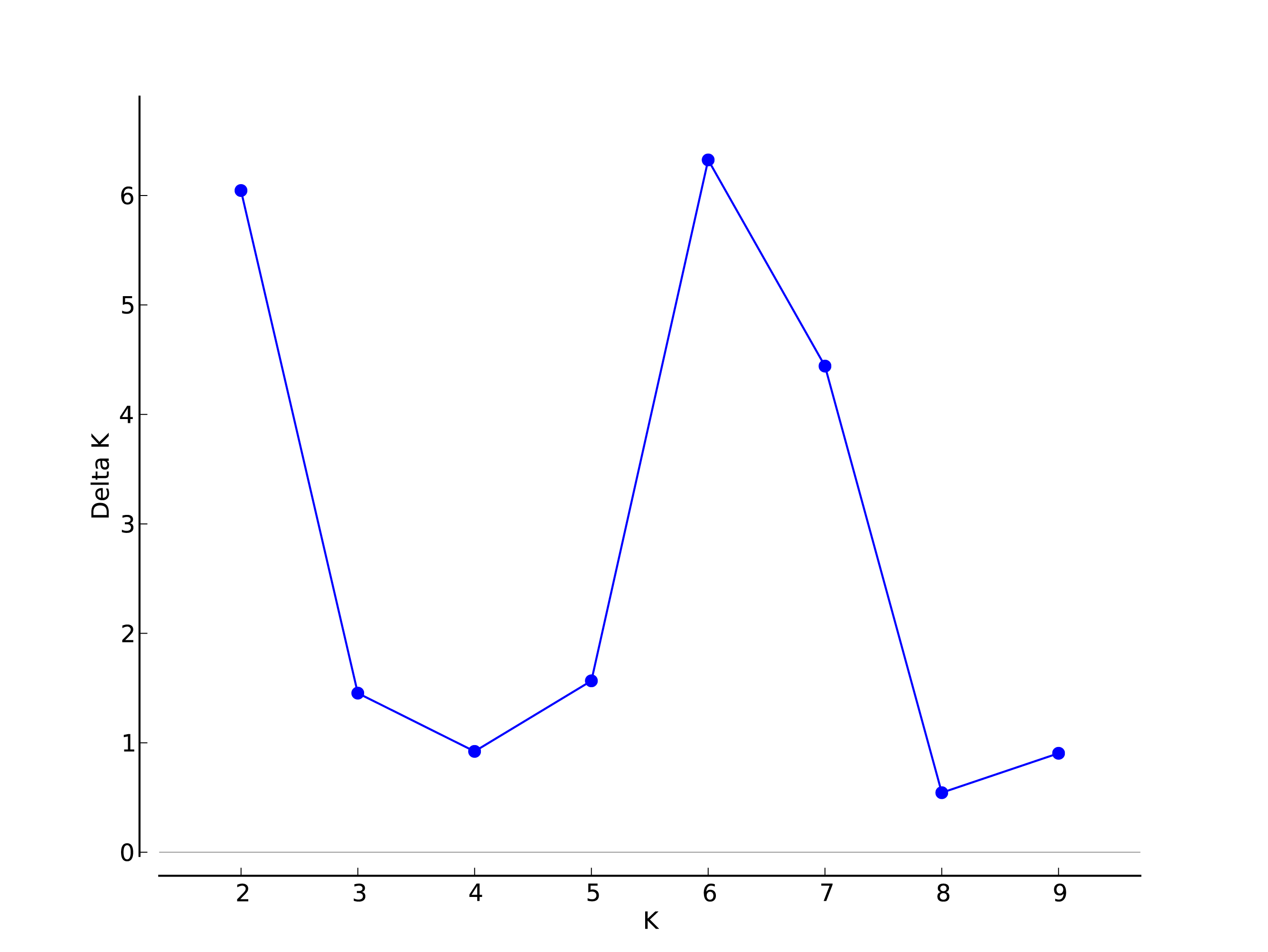


**Figure s2 | The optimal *K* value with the most suitable population clusters determined according to the values of *ΔK*.** *ΔK* was estimated by Structure Harvester.


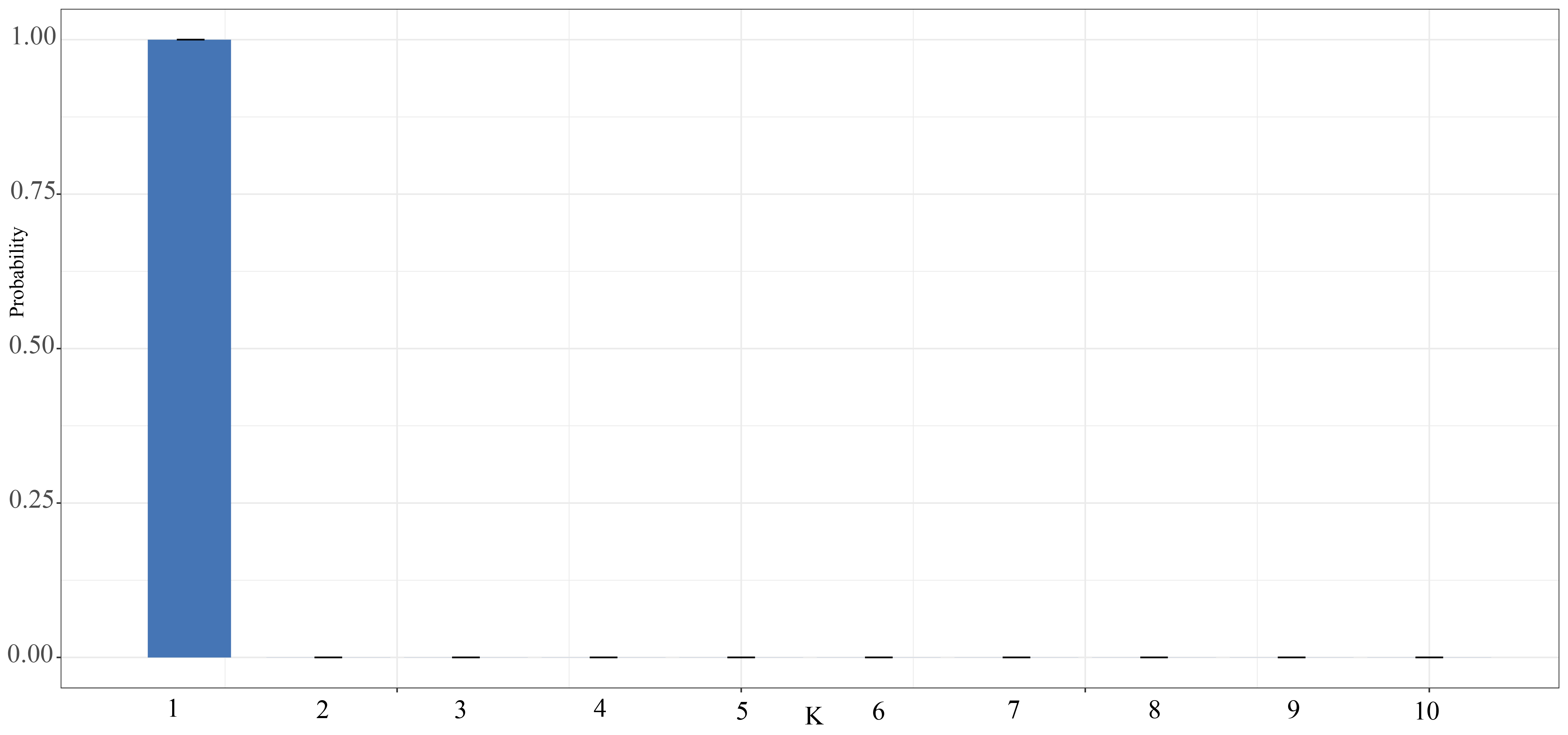


**Figure S3 | The options for estimating *K* according to the value of TI posterior probability by RmavericK.**
